# High-throughput screening identification of novel immunomodulatory combinations for the generation of tolerogenic dendritic cells

**DOI:** 10.3389/fmed.2023.1298424

**Published:** 2024-01-05

**Authors:** Sihan Jia, Jeremiah Kim, Aaron Palmer Esser-Kahn, Peter Deak

**Affiliations:** ^1^Chemical and Biological Engineering Department, Drexel University, Philadelphia, PA, United States; ^2^Pritzker School of Molecular Engineering, University of Chicago, Chicago, IL, United States

**Keywords:** dendritic cell, tolDC, tolerance, high-throughput screening (HTS), antigen-specific T_reg_, T_reg_

## Abstract

**Introduction:**

Tolerogenic Dendritic Cells (tolDCs) have an exceptional promise as a potential therapy for autoimmune disease and transplantation rejection. TolDCs are a unique phenotype of antigen presenting cells (APCs) that can influence naïve T cells into antigen specific T regulatory cells (T_regs_), which can re-establish tolerance against auto/allo-antigens in the long term. Despite their promise, tolDCs have not found clinical success. Most strategies seek to generate tolDCs *ex vivo* by differentiating naïve dendritic cells (DCs) with immunosuppressive agents. Recently, we developed a tolDC generation strategy, which we call Push/Pull Immunomodulation (PPI). In PPI, DCs are treated with combinations of toll-like-receptor (TLR) agonists and immunomodulatory agents, which generate more robust, T_reg_-inducing tolDCs than previous strategies. Here, we seek to identify more potent and clinically viable PPI formulations using data from a high-throughput screening project.

**Methods:**

Over 40,000 combinations of pathogen-associated molecular patterns (PAMPs) and immunomodulatory small molecules were screened using a modified murine macrophage line, RAW dual cells, to observe the effect of these combinations on two major immune regulatory transcription factors, NF-κB and IRF. Combinations were further screened for inflammatory cytokine activity using a human monocyte cell line, THP-1, then on murine DCs. Leading candidates were co-cultured with T cells to assess antigen specific T cell responses.

**Results:**

From this data, we identified 355 combinations that showed low or moderate IRF activity, low NF-κB activity, low inflammatory cytokine generation and good viability: all hallmarks of tolerogenic potential. We further screened these 355 combinations using bone marrow derived DCs (BMDCs) and identified 10 combinations that demonstrated high IL-10 (tolerogenic) and low TNF-α (inflammatory) secretion. After further optimizing these combinations, we identified two combinations that generate robust tolDCs from BMDCs *ex vivo*. We further show that these PPI-tolDCs can also generate antigen specific T_regs_ but do not increase overall T_reg_ populations.

**Discussion:**

These second-generation PPI formulations have significant potential to generate robust tolDCs and strong antigen specific T_regs_.

## Introduction

A proper balance between inflammatory and tolerance processes in immunity is essential for healthy immune function. Disruptions of this balance lead to autoimmune diseases. Therapies that can restore immune balance without generating widespread immune suppression are a major challenge for autoimmune therapies. Prior therapies like immunosuppressant drugs, such as dexamethasone, that broadly suppress immune function have been used for years to treat autoimmunity and transplant rejection (1). While advances in this class of non-selective immune suppressants have made them less harmful, they still cause side effects and increase the risk of cancer and infection (2). Even more selective targeted therapies, such as monoclonal antibodies that block selected immune receptors, bind a myriad of immune cell types presenting the targeted receptor and have broad immunosuppressive characteristics (3). These immunosuppressive regimens have been highly refined over the years for autoimmunity and have greatly improved the quality of life for many patients. Nevertheless, these therapies require constant maintenance doses and fundamentally do not eliminate the underlying auto-/allo-antigen-specific immunity.

Restoring proper balance between immunity and tolerance requires more subtle and engineered tools. One novel method co-opts an immune cell responsible for maintaining this balance, tolerogenic dendritic cells (tolDCs). Dendritic cells (DCs) are innate immune cells responsible for presenting foreign antigens to naïve T cells to generate antigen-specific immunity in the form of effector T and B cells (4). TolDCs are a subtype of DCs that express more tolerogenic markers, such as PD-L1, and secrete immunosuppressive cytokines, such as TGF-β and IL-10, while downregulating inflammatory markers such as CD80, DC maturation markers such as CD40, and inflammatory cytokines such as TNF-α and IL-6 (5). TolDCs have a multifaceted influence over adaptive immunity. TolDCs suppress local T-cell proliferation and promote anergy or deletion (6). TolDCs similarly can present antigens to naïve T cells but generate T regulatory cells (T_regs_), which suppress immunity in an antigen-specific fashion (5). TolDCs arise naturally, and there is a large body of literature on strategies to induce endogenous tolDC, but to date, none of these therapies have been clinically approved (5).

An orthogonal and perhaps more straightforward cell therapy strategy is *ex vivo* differentiation of induced tolDCs (itolDCs). Here, naïve DCs are generated from patient bone marrow or, more commonly, from monocyte-derived DCs (moDCs), treated *ex vivo* with immunosuppressive or immunomodulatory molecules, and reintroduced into the patient with an antigen of interest against an autoimmune disease (7). ItolDC therapies have shown great promise clinically for both autoimmunity and transplantation. It has been shown clinically that itolDC infusions can reduce inflammatory markers, alleviate symptoms of autoimmune disease, or prevent transplant rejection (8, 9). There is also strong evidence that itolDCs can generate persistent antigen-specific T_regs_ (10). One study from Nikolic et al. found that type 1 diabetes patients injected with itolDCs with proinsulin antigen maintained antigen-specific T_regs_ for 3 years, although this was only observed in three of the eight patients (8). The next goal for itolDC therapies is to improve the reliability and persistence of tolDCs, T_reg_ responses, and ultimately the prevention of unwanted immunity without the need for chronic immunosuppression.

Most itolDC strategies utilize a small number of immunosuppressive drugs, such as dexamethasone, or immunomodulators, such as vitamin D3, rapamycin, and IL-10 (11). Most clinical itolDC formulations further use lipopolysaccharide (LPS) or tumor necrosis factor-alpha (TNF-α) as a maturation agent after treatment with immunosuppressant/immunomodulatory drug to facilitate proper antigen presentation (12). While these formulations have shown success, as previously highlighted, none are currently clinically approved or used as a standard of care for autoimmunity or transplantation. There is a need to both expand the potential pool of itolDC induction agents and improve the strategy of *ex vivo* itolDC differentiation to improve their ability to facilitate T_reg_ responses.

In a previous publication, we identified that synergistic combinations of immunomodulatory drugs with immune agonists generated more robust and proliferative tolDCs. We showed that this combination treatment, which we call push/pull immunomodulation (PPI), could generate more antigen-specific T_regs_ than immunosuppressive or immunomodulatory compounds alone (13). PPI is the first example of using synergistic combinations of immune agonists/modulators co-delivered to naïve DCs to generate tolDCs (13). This preliminary PPI formulation was complex; it utilized three modulators and two agonists, limiting its clinical potential despite its ability to generate robust antigen-specific T_regs_.

Our goals for this study are to (1) use a large screening process to identify additional PPI pairs and (2) validate combinations that promote antigen-specific T_reg_ responses. In that effort, we utilized previously published data from a large cellular screen of over 40,000 immune agonists and small molecules to identify 355 novel, non-inflammatory combinations (14). We screened these 355 combinations with bone marrow-derived DCs (BMDCs) and selected 10 compounds that generated high IL-10/TNF-α ratios, indicating tolDC phenotypes. We then optimized these PPI combinations and identified two novel combinations that generate highly effective tolDCs (PPI-tolDCs), which, in turn, generate strong antigen-specific T_reg_ populations without increasing overall T_regs_.

## Materials and methods

### Materials

All fluorescently tagged antibodies, αCD28/CD3 antibodies, ELISA kits, RBC lysis buffer, Foxp3/Transcription Factor Staining Buffer Set, EDTA, and Cell Proliferation Dye eFluor^™^ 670 were purchased from Invitrogen. All inhibitors (modulators) were purchased from MedChemExpress. Bovine serum albumin (BSA) was purchased from VWR Life Science. HBSS, DPBS, PBS, DMEM, RPMI 1640, AIM-V medium, FBS, HI-FBS, HEPES, and non-essential amino acid solutions were purchased from Gibco. RAW-Dual cells, RAW Blue cells, QB buffer and reagent, EndoFit Ovalbumin (OVA), ODN 1826 (CpG), and all other TLR agonists were purchased from InvivoGen. Spleen Dissociation Medium and EasySep Mouse T-Cell Isolation Kit were purchased from STEMCELL Technologies. β-Mercaptoethanol was purchased from MP Biomedicals. Cell Activation Cocktail (without Brefeldin A), Recombinant Mouse GM-CSF (carrier-free) (20 ng/mL), and LEGENDplex MU Th1/Th2 Panel (8-plex) Kit were purchased from BioLegend. ProT2 MHC Class II tetramers and Pro5 MHC Class I pentamers were purchased from ProImmune. All plates, unless noted otherwise, were purchased from Thermo Fisher Scientific.

### NF-κB and IRF transcription factor screening

RAW-Dual cells (InvivoGen) were plated at 50,000 cells per well in 45 μL of DMEM with 5% HI-FBS from col 2 to 23 in clear flat-bottom 384 well plates (Greiner Bio-One). Cells attached at room temperature for 1 h. Fifty nanoliter of 10 mM modulator libraries were added by JANUS G3 via pintool to experimental wells (cols 3–22) for a final concentration of 10 μM. Following 1 h incubation, 5 μL of PRR agonist was added via a MultiDrop Combi liquid handler (col 3–23). Cells were incubated at 37°C and 5% CO_2_ overnight. Twenty hours later, 12.5 μL of QUANTI-Luc Plus was plated in an opaque, white 384-well plate. Five microliter of RAW-Dual cell supernatant was then added via MultiDrop Combi liquid handler before measuring luminescent values on a BioTek Synergy Neo2 plate reader as soon as the plate was completed. QUANTI-Luc Plus contains a stabilizer that reduces signal decay, allowing for comparable values throughout the read. In parallel, 15 μL of 5× concentrated QuantiBlue was added directly to the remaining cell supernatant in the RAW-Dual cell plate. Absorbance values were measured at varying time intervals at 620 nm. These samples were incubated so that the PRR agonist control reported an absorbance signal of approximately 1 A.U.

### Viability monitoring

Viability was monitored after overnight modulator addition by monitoring confluency via IncuCyte imaging. Two sets of parameters were used to create a confluency mask over all imaged wells. Modulators were considered toxic if both sets of confluency masks were <70% of those of resting cells. Confluency masks were quantified using IncuCyte software. This methodology was validated with select library plates using a traditional CellTiter Glo assay (Promega).

### Tertiary cytokine level screening

THP-1 cells were seeded at 50,000 cells per well in 45 μL of biotin-free RPMI +5% HI-FBS in clear 384-well plates. Following 24 h incubation, 50 nL of 10 mM modulator libraries were added by JANUS G3 via pintool to experimental wells (cols 3–22) for a final concentration of 10 μM. After 1 h, 5 μL of agonist was added via a MultiDrop Combi liquid handler (col 3–23). The following day, 5 μL of supernatant was transferred to white, low-volume ProxiPlates (PerkinElmer). According to the AlphaPlex protocol, 10 μL of a prepared acceptor bead (10 μg/mL final conc.) and biotinylated antibody (1 nM final conc.) mixture was added via liquid handler to the cell supernatant. After 1 h incubation at RT, 5 μL of donor beads (40 μg/mL final conc) were added in the dark. After an additional 1 h incubation, plates were read on a BioTek Synergy Neo2 plate reader with AlphaPlex filters for europium (615 nm) and terbium (545 nm) emission. A separate plate was run each day, containing a standard curve of a known analyte for interpolation purposes.

### Cell culture

All cells used in this study were cultured at 37°C and 5% CO_2_ in an incubator. THP-1 cells were cultured with RPMI 1640 (Life Technologies), 10% FBS (Sigma-Aldrich), and split every 2–3 days. RAW Blue cells (Invitrogen) were cultured in DMEM and 10% FBS and split every 2–3 days.

### BMDC cell culture

Bone marrow was harvested from 6 weeks-old C57BL/6 mice and differentiated into dendritic cells (BMDCs) using supplemented culture medium: RPMI 1640 (Life Technologies), 10% HI-FBS (Sigma-Aldrich), Recombinant Mouse GM-CSF (carrier-free) (20 ng/mL; BioLegend), 2 mM l-glutamine (Life Technologies), 1% antibiotic-antimycotic (Life Technologies), and 50 μM β-mercaptoethanol (Sigma-Aldrich). Bone marrow cells were treated with ACK lysis buffer for 10 min, washed, and plated with differentiation media at 1 million cells in 10 mL culture media. Cells were used for experiments on days 5–9.

### IL-6/IL-10 secondary screen

After 6 days of culture, BMDCs were plated at 100,000 cells per well in 200 μL and incubated overnight at 37°C and 5% CO_2_. Cells were treated with a modulator (10 μM). After 1 h, an agonist was added. Cells were incubated for 4 h at 37°C and 5% CO_2_. Supernatant cytokines were measured using the ELISA IL-6 and IL-10 kits (Thermo Fisher Scientific) according to the standard protocol. Supernatant measurements were performed in biological triplicates using cells from separate mice. Data were acquired on a BioTek Synergy Neo2 plate reader and analyzed via GraphPad Prism.

### RAW Blue NF-κB assay

RAW Blue cells were placed in a 96-well flat-bottom culture plate (Falcon) at 100,000 cells per well in 200 μL with DMEM and 10% HI-FBS and were incubated overnight at 37°C and 5% CO_2_. Cells were then treated with the top 10 PPI combinations (concentrations indicated in Table 1) and incubated overnight at 37°C and 5% CO_2_. For each sample, about 20 μL of the cell supernatant and 180 μL of freshly prepared QuantiBlue solution were combined in a new 96-well flat-bottom plate and incubated for 1 h at 37°C and 5% CO_2_. The plate was analyzed by placing it under a Multiskan FC plate reader. Absorbance was measured at 620 nm.

**Table 1:** Top 10 compounds identified from tertiary screen in Figure 1.

PPI	Top compounds	MW	Concentration (μM)	Agonist	Concentration (μg/mL)
1	Cucurbitacin I	514.65	10	LPS	0.1
2	Costunolide	232.32	10	Pam2	0.1
3	MLN120B	366.80	10	Pam2	0.1
4	(−)-Parthenolide	248.32	10	Pam2	0.1
5	Peficitinib	326.39	1	Pam2	0.1
6	Oclacitinib maleate	453.51	1	R848	12.5
7	AD80	473.43	1	Pam2	0.1
8	Cucurbitacin B	558.70	1	R848	12.5
9	CEP-33779	462.57	1	R848	12.5
10	Dehydrocostus lactone	230.30	1	Pam2	0.1

### Flow cytometry

Flow cytometry was performed on an Attune NxT Acoustic Focusing Cytometer (two-laser, seven-channel) for all experiments except for T-cell stimulation analysis using an immunophenotyping panel with OVA-specific tetramers, which was performed on the BD FACSymphony A5 (5-laser, 23-color). In a 96-well V bottom plate, 1 million cells were stained per sample with mouse CD16/CD32 monoclonal antibodies and incubated in the fridge for 20 min. Then cell surface markers with corresponding antibodies were stained and incubated in the fridge for 1 h. All staining steps were performed in the dark. All antibodies were diluted in a stain solution containing DPBS, 1% BSA (10 mg/mL), and 0.1 mM EDTA. After staining, cells were washed two times, placed in a fresh stain solution, and analyzed by flow cytometry unless otherwise noted. Samples were gated on FSC and SSC for live and single cells. Compensations were applied to samples by single staining. All raw data were analyzed by FlowJo software.

### *In vitro* validation of PPI-treated BMDCs

BMDCs were placed in 96-well flat-bottom culture plates (Falcon) at 200,000 cells per well in 200 μL using BMDC-supplemented culture medium on days 5 and 10 and were incubated overnight at 37°C and 5% CO_2_. Cells were then treated with the top 10 PPI combinations. The modulator and inhibitor ratios of the top 10 PPI combinations identified in Table 1 were altered to generate four different PPIs (1:1, 0.1:1, 1:0.1, and 0.1:0.1 modulator-to-agonist ratio). PPIs were incubated with BMDCs for 24 h at 37°C and 5% CO_2_. BMDCs were then tested for IL-10 and TNF-α via ELISA and for CD40, CD80, CD11c, and PD-L1 via flow cytometry.

### CpG rechallenge

Similar to *in vitro* validation of PPI-treated BMDCs, after incubation with PPIs for 24 h, BMDCs were washed two times with BMDC-supplemented culture medium and incubated with 0.5 μM CpG for 24 h at 37°C and 5% CO_2_. Then they were tested for IL-10 and TNF-α via ELISA and for CD40, CD80, CD11c, and PD-L1 via flow cytometry.

### ELISA

TNF-α and IL-10 levels were tested by commercial kits from Invitrogen. TNF-α supernatants were analyzed 4 h after PPI incubation or CpG rechallenge. IL-10 supernatants were analyzed 24 h after PPI incubation or CpG rechallenge. The data were measured on a Multiskan FC plate reader.

### Splenocyte extraction

Spleens were harvested from 6 weeks-old C57BL/6 female mice dissected into 1 mm-sized portions and placed in commercially purchased Spleen Dissociation Medium (STEMCELL Technologies) for 30 min at 37°C and 5% CO_2_. Then the splenocytes were passed through a 70 μm filter, centrifuged at 200 × *g* for 5 min, and resuspended in HBSS +2% HI-FBS + 1 mM EDTA.

### T-cell isolation

Murine T cells were isolated from extracted splenocytes using the EasySep Mouse T-Cell Isolation Kit (STEMCELL Technologies). After being isolated by the magnetic kit, T cells were treated with RBC lysis buffer (Invitrogen) for 5 min at room temperature before final use.

### T-cell proliferation assay

Isolated T cells were washed with HBSS two times before staining with Cell Proliferation Dye eFluor^™^ 670 (Invitrogen). T cells were resuspended in HBSS and stained with 1:1,000 diluted proliferation dye for every 10 million cells at 37°C and 5% CO_2_ for 10 min. Then T cells were treated with 10% HI-FBS at room temperature for 5 min to stop staining, washed two times with HBSS +2% HI-FBS + 0.5 mM EDTA, and resuspended in T-cell culture media (Aim V + 10% HI-FBS + 1 mM HEPES +1× non-essential amino acid solutions +50 μM β-mercaptoethanol). T cells were placed in 24-well flat-bottom culture plates (Falcon) at 800,000 cells per well in 1 mL and combined with 200,000 cells per well from the top 8 PPI-treated BMDCs at a ratio of 4:1 (T cells to BMDCs). In the non-specific assay, including controls of blank (T cells only), positive (T cells +0.5 μL/mL T-cell stimulation cocktail with PMA/ionomycin (BioLegend) + untreated BMDCs), DCs treated with 12.5 μg/mL R848 (R848 DC), and naïve DC (untreated BMDCs). All groups contained T cells, and all groups except blank were treated with 100 ng/mL αCD28/CD3. In the OVA-specific assay, including controls of blank (T cells from OVA-vaccinated mice only), positive [T cells from OVA-vaccinated mice + naïve BMDCs +0.5 μL/mL T-cell stimulation cocktail with PMA/ionomycin (BioLegend)], R848 DC (OVA-vaccinated T cells + DCs treated with 12.5 μg/mL R848) and naïve DC (T cells from OVA-vaccinated mice + naïve BMDCs). All groups except blank were treated with 10 μg/mL OVA. Cells were incubated at 37°C and 5% CO_2_ for 72 h. Supernatants were analyzed for cytokine secretion using the LEGENDplex MU Th1/Th2 Panel (8-plex) Kit (BioLegend). Cells were stained for CD3, CD4, CD8, and CD69 and analyzed by flow cytometry.

### T_reg_ assay

Similar to the T-cell proliferation assay, after washing two times with HBSS, isolated T cells were directly combined with PPI-treated BMDCs at a ratio of 4:1 (T cells to BMDCs) and incubated for 72 h at 37°C and 5% CO_2_. Cells were first stained with surface markers CD4, CD8, and CD25. Then Foxp3/Transcription Factor Staining Buffer Set (Invitrogen) was used, and standard protocols were followed to stain Foxp3. All markers were analyzed via flow cytometry.

### LEGENDplex cytokine analysis

In the T-cell proliferation assay, after 72 h incubation, supernatants were analyzed for cytokine secretion using the LEGENDplex MU Th1/Th2 Panel (8-plex) Kit (BioLegend) according to the standard protocol. The data were analyzed via flow cytometry.

### Live animals

Six weeks-old C57BL/6 female mice were housed and treated according to the approved IACUC protocol by Drexel University Laboratory Animal Resources.

### OVA mouse vaccination

C57BL/6 mice were vaccinated with 100 μg of OVA and 50 μg of the immune agonist CpG per mouse in 100 μL by IP injection. After waiting for 10 days, murine spleens were harvested for extracting splenocytes.

### Immunophenotyping panel flow analysis

Similar to the T_reg_ Assay, after washing two times with HBSS, isolated T cells were directly combined with PPI-treated BMDCs at a ratio of 4:1 (T cells to BMDCs) and incubated for 72 h at 37°C and 5% CO_2_. In the OVA-specific assay, cells were washed two times with a stain solution containing DPBS +1% BSA (10 mg/mL) + 0.1 mM EDTA before being stained with 50 μL staining solution +5 μL ProT2 MHC Class II tetramers +10 μL Pro5 MHC Class I pentamers (ProImmune) per sample at 37°C and 5% CO_2_ for 30 min. Without washing, each sample was stained with 2 μL mouse CD16/CD32 monoclonal antibodies on ice for 30 min. Then each sample was stained with 60 μL antibody cocktails containing surface markers of CD8, CD3, CD69, CD19, CD127, CD4, CD25, CD27, CD44, CTLA-4 (CD152), CD28, PD-1 (CD279), and CD45 in the fridge for 1 h. The cells were then washed one time with stain solution, used Foxp3/Transcription Factor Staining Buffer Set (Invitrogen), and followed standard protocols to stain Foxp3. After washing two times with stain solution, each sample was resuspended in 200 μL stain solution and tested by flow cytometry using BDFACS Symphony A5. The unspecific assay was identical to the OVA-specific assay, except that tetramers and pentamers were not stained. Samples were compensated based on single staining. All raw data were analyzed by FlowJo.

### Statistical analysis

Statistical significance was determined using GraphPad Prism (version 10.0). All experiments were carried out in biological triplicates, and *p*-values <0.05 were determined to be significant. *p*-values are listed in the figures. *p*-values were determined by one-way ANOVA with Tukey *post hoc* test or two-tailed student’s *t*-test for single comparisons, with the test used listed in the figure legend.

## Results

### Identification of potential PPI candidates from large cellular screen

**Figure 1:**
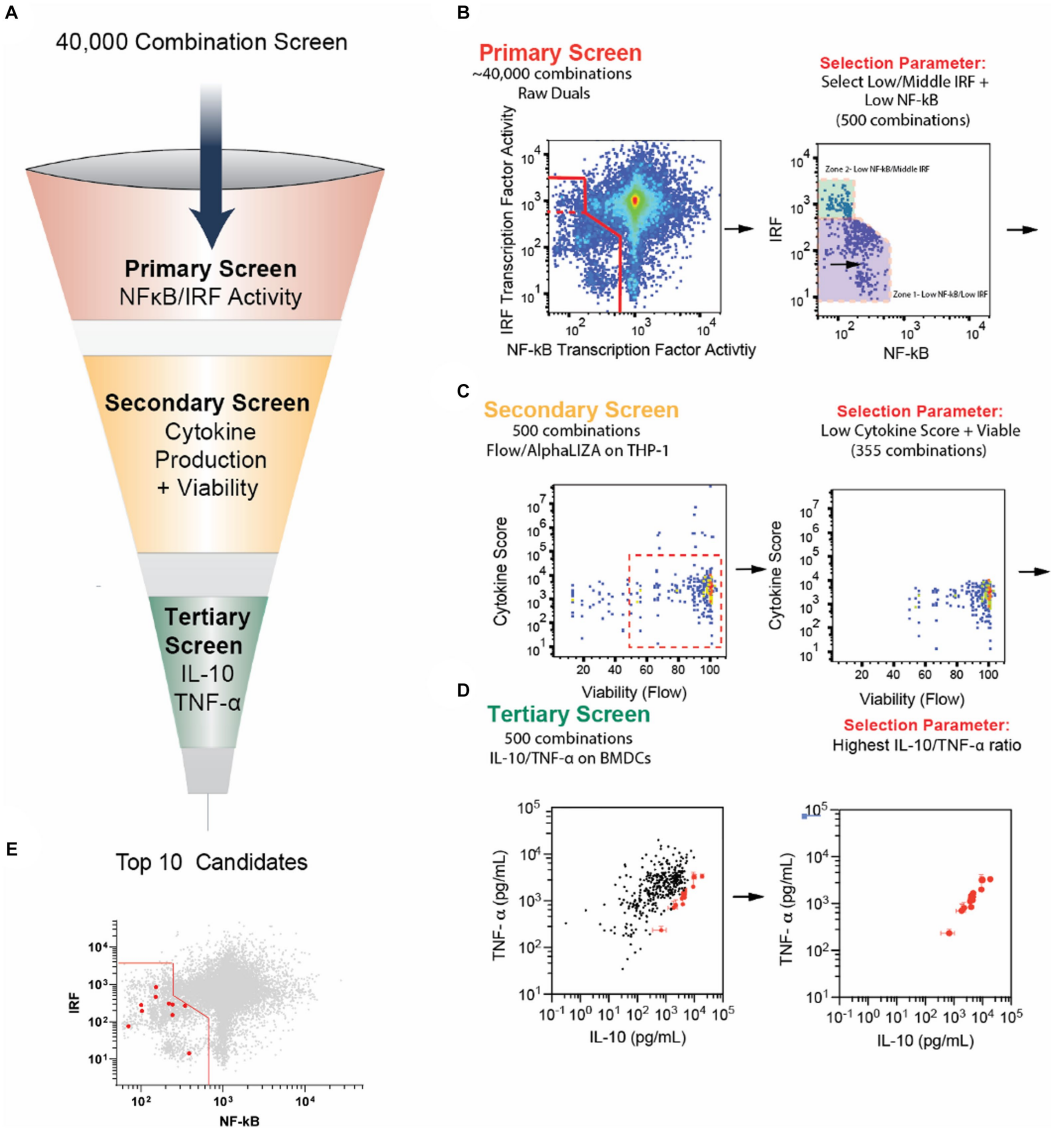
Selection of new PPI candidates using high-throughput screening. **(A)** Schematic of screening process to evaluate PPI candidate combinations. **(B)** Primary screening results. Forty thousand combinations of small molecule modulators and agonists were tested on RAW-Dual cells to observe the effects on IRF and NF-κB activity. Combinations with low NF-κB activity and mid- to low IRF activity were chosen for a total of 500 combinations. **(C)** Secondary screening results. The 500 combinations from the primary screen were tested on THP-1 human monocytes overnight. Cell viability was monitored via microscopy, and the secretion of cytokines and chemokines, TNF-α, IL-6, IL-1β, IL-12, IP-10, CCL-4, and IFN-β, was monitored with AlphaLISA. Combinations with <50% viability were removed. Cytokine secretion was normalized to agonist-only control, multiplied by 1,000, and all six normalized scores were summed to generate a “cytokine score.” Combinations with cytokine score >6,000 (indicating an increase in overall inflammatory cytokines) were removed. **(D)** The remaining 355 combinations were tested on BMDCs for 24 h, and the secretion of IL-10 and TNF-α was measured using standard ELISA. The top 10 combinations with the highest IL-10/TNF-α ratio are highlighted in red. **(E)** Top 10 combinations are highlighted in red from the graph in part B. Note that the data for parts **(B–D)** were a re-analysis of data originally obtained from a study by Kim et al. (14). Error bars in part E represent the ±SD of triplicate measurements. See the methods section for more information on the screening process.

To identify new PPI candidate combinations, we mined data from a previously published large cellular screen using combinations of immune agonists and small molecule drugs (Figure 1A) (14). The high-throughput screening examined levels of innate immune cell NF-κB and IRF activity, two important transcription factors that serve as proxies for cellular inflammatory responses and antigen presentation, respectively (14). It has been shown that tolerating responses have low NF-κB and low IRF activity, so we sought to use these data to identify potential PPI candidates (15, 16).

In the preliminary screen, 3,141 small molecule inhibitors were screened against 13 PRR agonists, with the majority being toll-like receptors, representing 40,833 unique combinations. The small molecule inhibitors were sourced from commercial screening libraries (Supplementary Table S1). NF-κB and IRF transcription factor activity were observed using the murine macrophage dual reporter cell line, RAW-Dual cells (Supplementary Table S2). Cells were incubated with agonists and modulators for 24 h, and the supernatant was drawn for simultaneous analysis, and relative IRF and NF-κB activity was plotted relative to agonist-only controls (Figure 1B).

After the primary screen, we selected the 500 modulator and agonist combinations that demonstrate low NF-κB and low or moderate IRF. While the low activity of both NF-κB and IRF has been correlated with tolerogenic cell phenotypes, we hypothesized that moderate IRF may help tolDC present antigen and retain a robust APC phenotype (15, 16). We normalized both NF-κB and IRF activity with respect to agonist-only controls and summed them. We selected the 400 combinations with the lowest sum of IRF and NF-κB. Additionally, we included an additional 100 combinations with the lowest NF-κB activity, not in the original 400. This group represented combinations with low NF-κB and moderate IRF activity (Figure 1B).

We next sought to remove candidate combinations that were cytotoxic or generated inordinate levels of inflammatory cytokines. To do this, we further mined data from a secondary screen used in the large screening study (14). Each of the 500 combinations was tested for cytokine secretion of the cytokines and chemokines IL-12, IL-6, IFN-β, IP-10, TNF-α, and CCL4 on THP-1 cells using an AlphaPlex assay and viability using IncuCyte imaging. We then removed compounds with high levels of cytokine secretion and low viability to further reduce our total combinations to 355 (Figure 1C). Next, to reduce the number of compounds to carry forward to *in vivo* studies, we performed a tertiary screen by incubating BMDCs with each combination and testing cytokine secretion. We selected the combinations with the highest IL-10 to TNF-α ratio, as these cytokines broadly represent tolerance and inflammation, respectively (Figure 1D) (17, 18). These top 10 combinations were the PPI candidates we optimized and investigated in the remainder of this study (Table 1; Figure 1E).

### Optimization of top 10 PPI candidates

**Figure 2:**
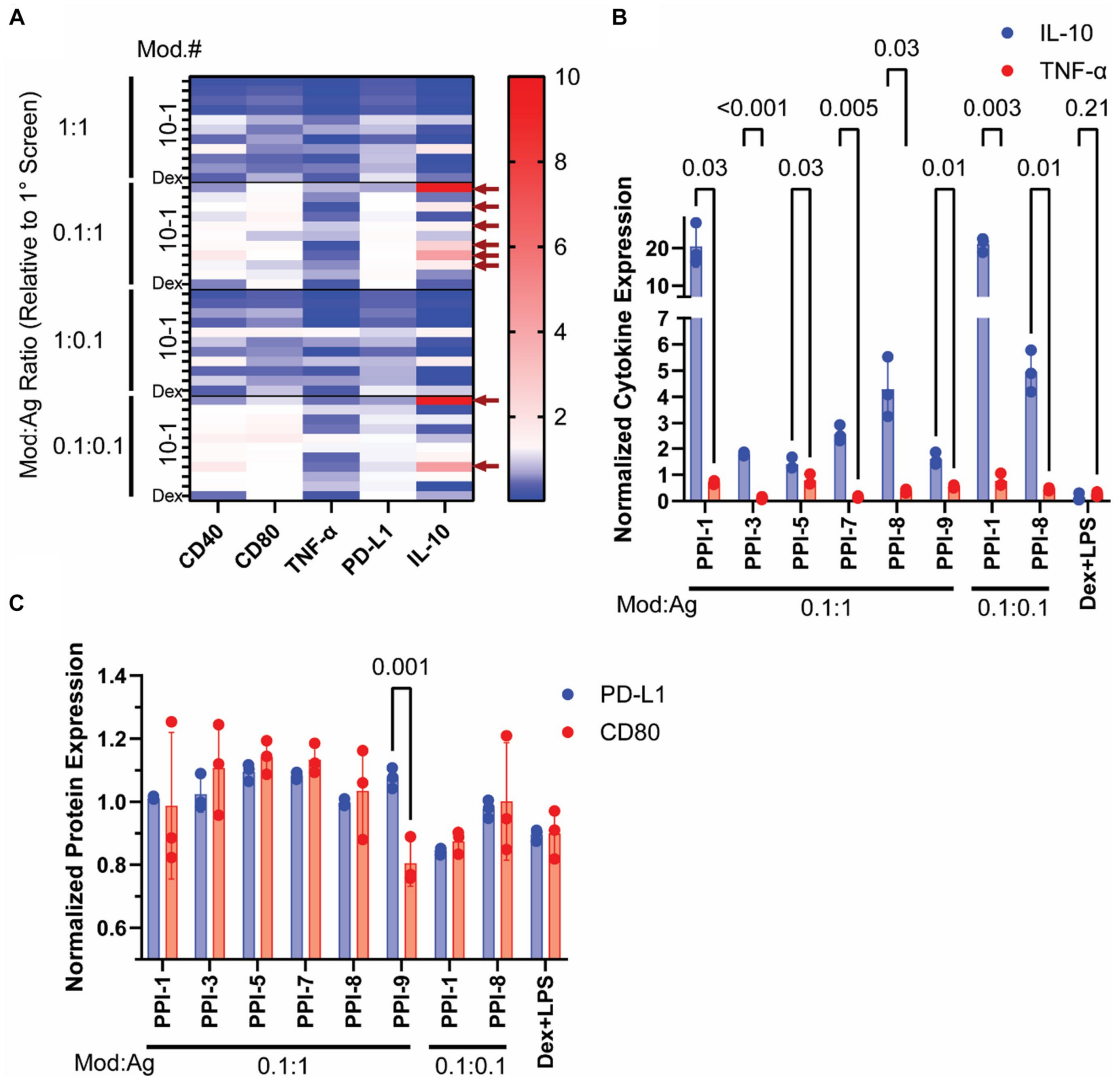
Optimization of PPI formulations. The modulator and inhibitor ratios of the top 10 PPI combinations identified in Figure 1 were altered to generate four different PPIs (1:1, 0.1:1, 1:0.1, and 0.1:0.1 modulator to agonist ratio compared to the original concentration identified via Figure 1 screening). PPIs were incubated with BMDCs on day 5 for 24 h and then tested for cytokine secretion via ELISA and surface protein expression via flow cytometry. **(A)** Heatmap of BMDC markers post-PPI treatment. The expression of CD80, CD40, TNF-α, PD-L1, and IL-10 was normalized to agonist-only controls and plotted. Blue indicates downregulation, white indicates unchanged, and red indicates upregulation from agonist-only controls. Top 8 PPI combinations are identified by the red arrows on the left side of the graph. Full dataset is found in Supplementary Figures S2, S3. **(B)** Cytokine Expression of top 8 PPI combinations. Normalized IL-10 (blue) and TNF-α (red) are plotted. **(C)** Protein expression of top 8 PPI combinations. Normalized PD-L1 (blue) and CD80 (red). Gating strategy is found in Supplementary Figure S4. Significance was determined by a two-tailed student’s *t*-test with *p*-values listed on graphs. All control samples were performed in duplicates, and experimental groups were performed in biological triplicates. Error bars are ±SD.

After identifying 10 promising PPI combinations with high IL-10/TNF-α ratios, we optimized them by varying modulator (mod) and agonist (ag) concentrations. From initial concentrations in Table 1, we tested four concentration variations: equal mod and ag as in Table 1 (1:1), 10-fold reduced mod (0.1:1 mod:ag), 10-fold reduced ag (1:0.1 mod:ag), and 10-fold reduced both (0.1:0.1 mod:ag). We also included dexamethasone (10 μM) with lipopolysaccharide (LPS, 0.1 μg/mL) as a positive control, as this combination (Dex + LPS) is frequently used to generate tolDCs *ex vivo* (7, 19). We analyzed these PPI combinations using RAW Blue cells to track the PPI effect on NF-κB transcriptional activity and observed only a modest decrease compared to agonist-only controls (Supplementary Figure S1) (20). We further incubated these PPIs with BMDCs as shown in Figure 1D and tested CD40, CD80, and PD-L1 via flow cytometry (Supplementary Figure S2) and IL-10 and TNF-α via ELISA (Supplementary Figure S3). We normalized these data to agonist-only controls and plotted them in a heatmap (Figure 2A). Using these data, we identified the top 8 PPIs that increase IL-10 and PD-L1 simultaneously but do not increase CD40, CD80, or TNF-α and were used for further study (Figure 2A and Supplementary Table S3). These top 8 optimized PPI combinations showed significant increases in normalized IL-10 compared to TNF-α secretion (Figure 2B). The effects of PPI were more moderate on surface markers. We observed a significant change in the normalized expression of PD-L1 to CD80 only for PPI-9 (0.1:1 mod:ag ratio, Figure 2C). The other top 8, while they did not significantly reduce CD80 from BMDCs, did not alter PD-L1 when compared to agonist-only controls (Figure 2C). Through these experiments, we identified eight optimized PPI combinations that generate increased IL-10, suppress TNF-α, and do not suppress PD-L1 expression. These results indicated that PPI can both suppress inflammatory markers and either improve or not alter the expression of tolerance markers.

### PPI-tolDCs effectively suppress T-cell function and induce T regulatory cells against OVA, but not against αCD3/CD28 stimulation

**Figure 3:**
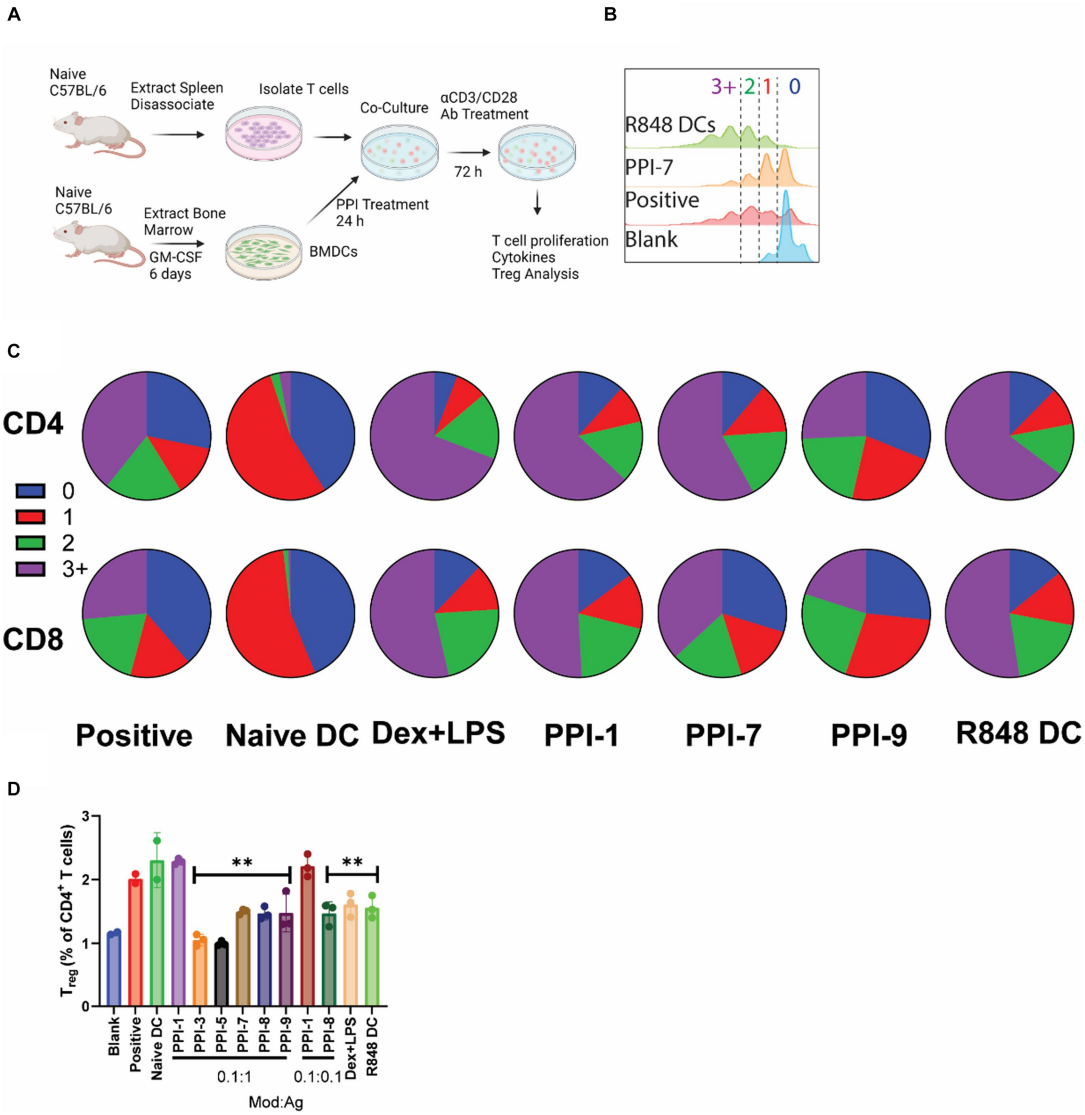
Non-specific PPI-tolDC-T-cell proliferation assay. **(A)** Schematic of non-specific T-cell stimulation experiments used for data in parts **(B–D)**. For each group, 800K T cells were incubated with 200K DCs and analyzed after 72 h. Controls included T cells only (blank), T cells + untreated DCs + 0.5 μL/mL T-cell stimulation cocktail with PMA/ionomycin (positive), untreated DCs (naïve DCs), and DCs treated with 12.5 μg/mL R848 (R848 DC). All groups contained T cells, and all groups except blank were treated with 100 ng/mL αCD28/CD3. **(B)** Representative flow plot of unspecific T-cell proliferation for CD4^+^ T cells with gating for generation number. T-cell proliferation by generation number for each treatment was calculated for part **(C)**. **(C)** Representative pie charts showing the breakdown of average T-cell generation for selected treatment groups. Top: CD4^+^ T cells Bottom: CD8^+^ T cells. Complete dataset can be found in Supplementary Figure S5. **(D)** Cells were further analyzed for CD4^+^ CD25^+^ FoxP3^+^ T cells (T_reg_), and the percentage of T_reg_ per CD4^+^ cells was calculated. Gating strategy is found in Supplementary Figure S7. Significance was determined by a two-tailed student’s *t*-test with Tukey post-hoc test. ** indicates *p* < 0.01. All control samples were performed in duplicates, and experimental groups were performed in biological triplicates. Error bars are ±SD.

While increasing positive markers of tolerance and decreasing markers of inflammation in BMDCs is important for demonstrating tolDC efficacy, the most critical measure of tolDC functionality is the downstream effect on T cells (21). To validate T-cell effects, we performed two different assays on the top 8 optimized combinations. In a non-antigen-specific assay, murine T cells were isolated from naïve C57BL/6 splenocytes using magnetic separation kits. In each well of a 96-well plate, 800K T cells were incubated with BMDCs that were previously treated with PPIs or LPS + Dex for 24 h prior and washed. T cells and DCs were co-incubated for 72 h with 100 ng/mL αCD28/CD3 antibodies to stimulate T-cell proliferation/activation (Figure 3A). Cells were then analyzed for T-cell proliferation, cytokine secretion, and the T_reg_ marker, FoxP3 (17). Controls included T cells only (blank), T cells + untreated DCs + 0.5 μL/mL T-cell stimulation cocktail with PMA/ionomycin (positive), DCs treated with 12.5 μg/mL R848 (R848 DC), and untreated DCs (naïve DCs). All groups contained T cells, and all groups except blank were treated with 100 ng/mL αCD28/CD3.

Under non-specific αCD3/CD28 stimulation, PPI-tolDC had no effect on suppressing either CD8^+^ or CD4^+^ T-cell proliferation and in some cases even induced additional division compared to positive controls (Figures 3A–C; Supplementary Figure S5). PPI-9 showed some suppression in CD8^+^ and CD4^+^ T-cell proliferation relative to positive control, but these were not statistically significant. All groups except PPI-3 and PPI-7 significantly suppressed CD69 expression compared to positive control, indicating suppression of T-cell activation (Supplementary Figure S5). Cytokine analysis showed increases in IL-10 and a reduction in TNF-α for most PPI groups relative to positive controls. All groups except PPI-1 showed reduction in IL-2 and IL-4 (with the exception of PPI-1, which had high IL-4), but interestingly, PPI-8 showed high levels of IL-6 and PPI-9 showed moderate levels of IL-6 (Supplementary Figure S6). We also observed that only PPI-1 generated a significant increase in T_reg_ (CD3^+^, CD4^+^, CD25^+^, and FoxP3^+^; see Supplementary Figure S8 for gating strategy) when compared to untreated controls (Figure 3D). These results suggest that most PPI treatments generate tolDCs, which do not induce broad T_reg_ populations and have more moderate or non-existent suppression of T-cell function when not specifically stimulated.

**Figure 4:**
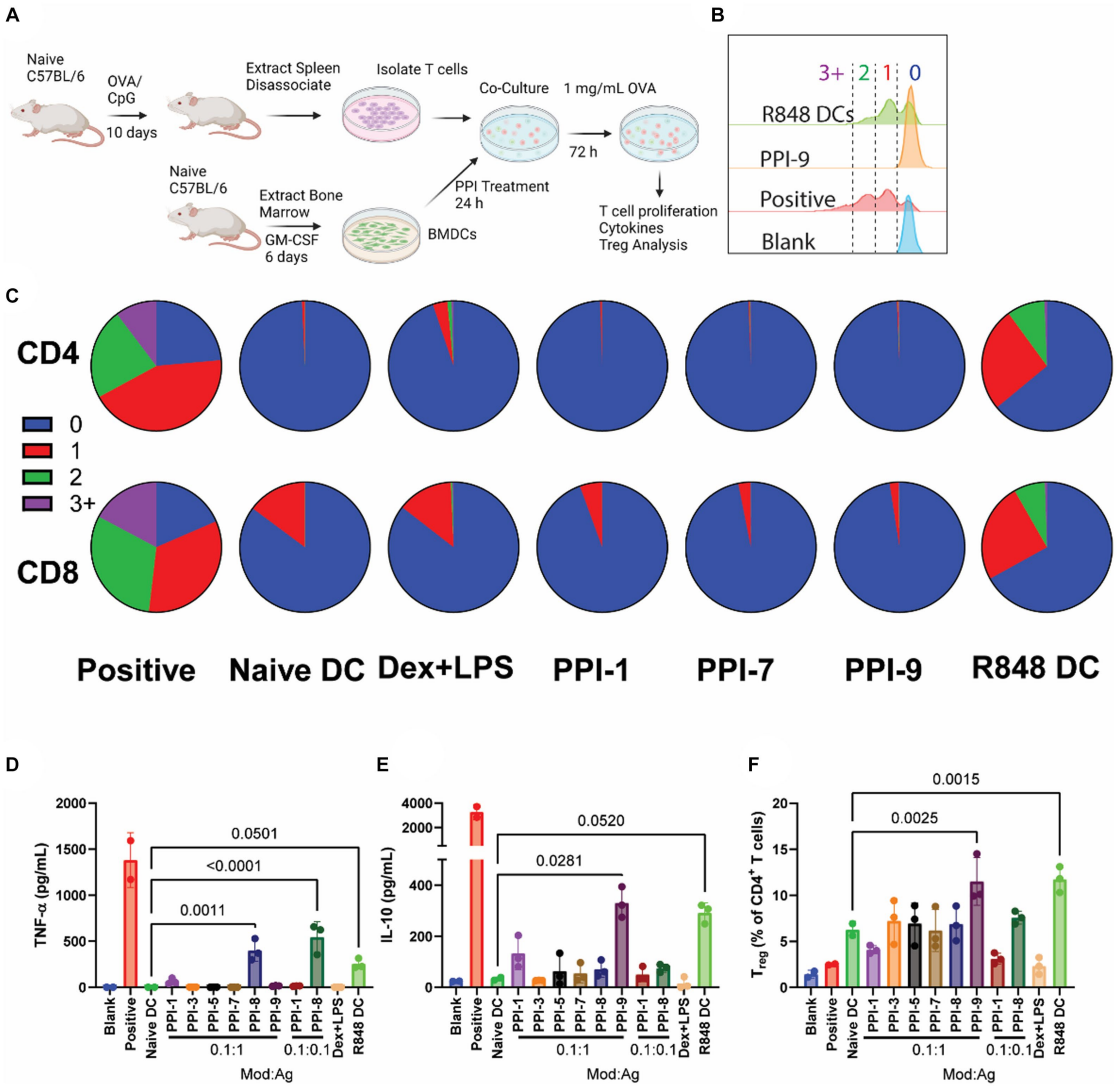
OVA-specific PPI-tolDC-T-cell proliferation assay. **(A)** Schematic of OVA-specific *ex vivo* T-cell stimulation experiment used for data in parts **(B–F)**. Here, 800K T cells from OVA-vaccinated mice were incubated with 200K DCs for each group in 2 mL of media. OVA stimulation was 1 mg/mL. T cells were analyzed after 72 h. Controls included T cells from OVA-vaccinated mice only (blank), T cells from OVA-vaccinated mice + naïve BMDCs +0.5 μL/mL T-cell stimulation cocktail with PMA/ionomycin (positive), and T cells from OVA-vaccinated mice + naïve BMDCs (naïve DC). All groups except Blank were treated with 10 μg/mL OVA. **(B)** Representative flow plot of OVA-specific T-cell proliferation for CD4^+^ T cells with gating for generation number. T-cell proliferation by generation number for each treatment was calculated for part C. **(C)** Representative pie charts showing the breakdown of average T-cell generation for selected treatment groups. Top: CD4^+^ T cells; bottom: CD8^+^ T cells. Complete dataset can be found in Supplementary Figure S5. **(D)** Supernatants were collected after 72 h and analyzed for IL-10 and **(E)** TNF-α secretion. **(F)** Cells were further analyzed for CD4^+^ CD25^+^ FoxP3^+^ T cells (T_reg_), and the percentage of T_reg_ per CD4^+^ cells was calculated. Gating strategy is found in Supplementary Figure S7. Significance was determined by a two-tailed student’s *t*-test with *p*-values listed on graphs. All control samples were performed in duplicates, and experimental groups were performed in biological triplicates. Error bars are ±SD.

We similarly tested PPI-tolDCs in an antigen-specific context using ovalbumin (OVA) as a model antigen. We vaccinated C57BL/6 mice with 100 μg of OVA and 50 μg of the immune agonist CpG1806 to stimulate OVA-specific immune responses (OVA-vaccinated mice, Figure 4A). Often, OVA responses are monitored using OT-1 and OT-2 mice, which have modified T-cell receptors to bind only to the major histocompatibility class I (MHC-I) or class II (MHC-II) epitopes of OVA (22). We, however, wanted a broader epitope diversity in the *ex vivo* T-cell stimulation assays and elected to generate immunity by vaccination (13). T cells isolated from OVA-vaccinated mice were then incubated with OVA (10 μg/mL) and PPI-treated BMDCs, isolated from naïve mice (Figure 4A). Controls included T cells from OVA-vaccinated mice only (blank), T cells from OVA-vaccinated mice + naïve BMDCs +0.5 μL/mL T-cell stimulation cocktail with PMA/ionomycin (positive), T cells from OVA-vaccinated mice + DCs treated with 12.5 μg/mL R848 (R848 DC), and T cells from OVA-vaccinated mice + naïve BMDCs (naïve DC). All groups except Blank were treated with 10 μg/mL OVA. T cells and DCs were co-incubated for 72 h, then analyzed by flow cytometry for T-cell proliferation and T_reg_ expression and by cytokine bead array for cytokines.

When analyzing OVA-stimulated T-cell proliferation, we observed that all tolDCs, including Dex + LPS, prevented T-cell proliferation compared to positive control over the course of 72 h for both CD8^+^ and CD4^+^ T cells (Figures 4A–C). This is expected for the naïve DC group, which lacks immune stimulation and therefore has low co-stimulatory molecule presentation, whereas the positive and R848 DC controls generated T-cell proliferation (Supplementary Figure S2). All PPI-tolDCs prevented T-cell division entirely; only Dex + LPS showed CD8 T-cell division in response to OVA stimulation (Figure 4C). All treated DC groups also suppressed CD69^+^ populations drastically when compared to positive control (1–3% vs. 70%, Supplementary Figure S5). All PPI formulations dramatically suppressed IL-2, and all except PPI-8 suppressed IL-4, TNF-α, and IFN-γ secretion (Supplementary Figure S6; Figure 4D). For cytokine secretion, only PPI-9 was able to generate IL-10 from T cells after antigen stimulation (Figure 4E). PPI treatment has a limited effect on overall T_reg_ populations; only PPI-9 showed any increase in T_reg_ populations, although the effect was insignificant when compared to naïve DC controls (Figure 4F).

While all PPI formulations did show some effect on tolerogenic adaptive responses, we elected to carry over three formulations (PPI-1, PPI-7, and PPI-9) at a 0.1:1 mod:ag ratio to additional *ex vivo* experiments. PPI-1 was chosen because of its robust IL-10 generation from treated DCs and its ability to generate T_reg_ with non-specific stimulation. PPI-7 was chosen due to its ability to reduce inflammatory cytokines/markers in T cells and suppress T-cell proliferation after both specific and non-specific activation. Finally, PPI-9 was chosen due to its capacity to increase PD-L1 on treated DCs, selectively increase T_regs_ after antigen stimulation, and generate IL-10 from T cells.

### PPI-tolDCs generate robust antigen-specific T_regs_ but do not increase overall T_reg_ populations

In a final series of *ex vivo* experiments, we wanted to observe how PPI-tolDCs generate antigen-specific T_reg_ responses and directly compare them to unspecific activation. We chose the top 3 PPI formulations (PPI-1, PPI-7, and PPI-9) to evaluate in these experiments. We repeated the two *ex vivo* T-cell stimulation assays from Figures 3, 4 and analyzed the T cells on a 20-color flow cytometry panel for more precise immunophenotyping. OVA-stimulated groups were also stained for the OVA major MHC-I epitope, OVA (257–264), and major MHC-II epitope, OVA (323–339), with OVA-stimulated T cells to identify antigen-specific T-cell populations.

**Figure 5:**
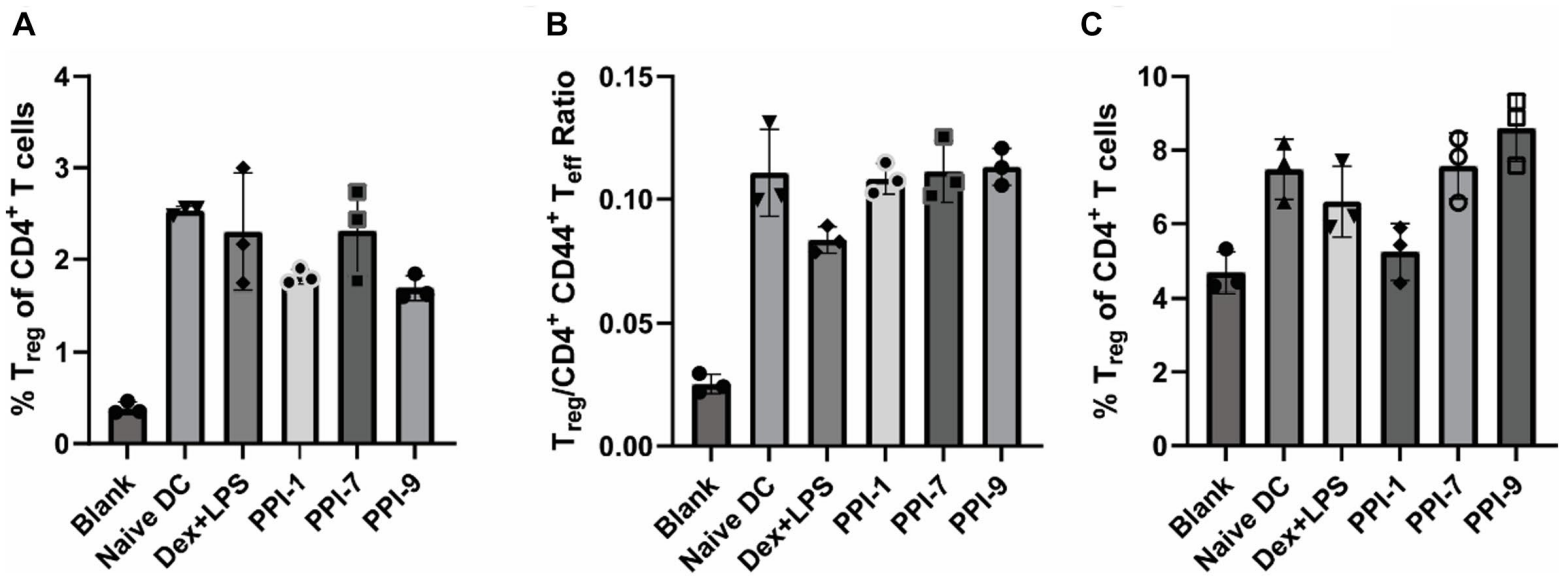
Immunophenotyping of PPI-tolDC activated T_regs_. **(A,B)** PPI-tolDCs were incubated with T cells stimulated with αCD3/CD28 (unspecific), as described in Figure 3, and analyzed with a large immunophenotyping panel (see Supplementary Figure S8 for gating strategy). Controls included naïve T cells only (blank) and naïve T cells with 100 ng/mL αCD3/CD28 and naïve DCs (Naïve DC). **(A)** Percentage of T_regs_ of CD4^+^ T cells. **(B)** Ratio of the total number of T_regs_ to CD44^+^ CD4^+^ T cells. **(C)** PPI-tolDCs were incubated with T cells from OVA-vaccinated mice and stimulated with OVA as in Figure 4 (specific) and analyzed with the same large immunophenotyping panel (see Supplementary Figure S8 for gating strategy). Controls included T cells from naïve mice with untreated DCs (Blank) and T cells from OVA treated mice with untreated DCs and 10 μg/mL OVA (Naïve DC). Plot shows the percentage of all T_regs_ of CD4^+^ T cells. All control samples were performed in duplicates, and experimental groups were performed in biological triplicates. Error bars are ±SD.

Upon analyzing αCD28/CD3 stimulated T cells co-incubated with treated DCs with a larger flow cytometry panel, we observed that PPI-tolDC and Dex + LPS treatment did not increase T_reg_ populations (CD3^+^ CD4^+^ CD44^−^ CD127^−^ CD25^+^ FoxP3^+^) when compared to naïve DCs or increase ratio of T effector (T_eff_, CD3^+^ CD4^+^ CD44^+^) cells, corroborating our results from Figures 3, 5A,B; see Supplementary Figure S8 for gating strategies. This indicated that PPI treatment did not broadly suppress immunity or non-specifically expand T_reg_ populations. It should also be noted that while the PPI-tolDCs did not alter cell viability and CD4/CD8 populations, Dex + LPS BMDCs significantly reduced CD4^+^ T-cell populations and total T-cell numbers, suggesting that Dex + LPS was highly and non-specifically suppressive, whereas PPI-tolDCs were not (Supplementary Figure S9).

**Figure 6:**
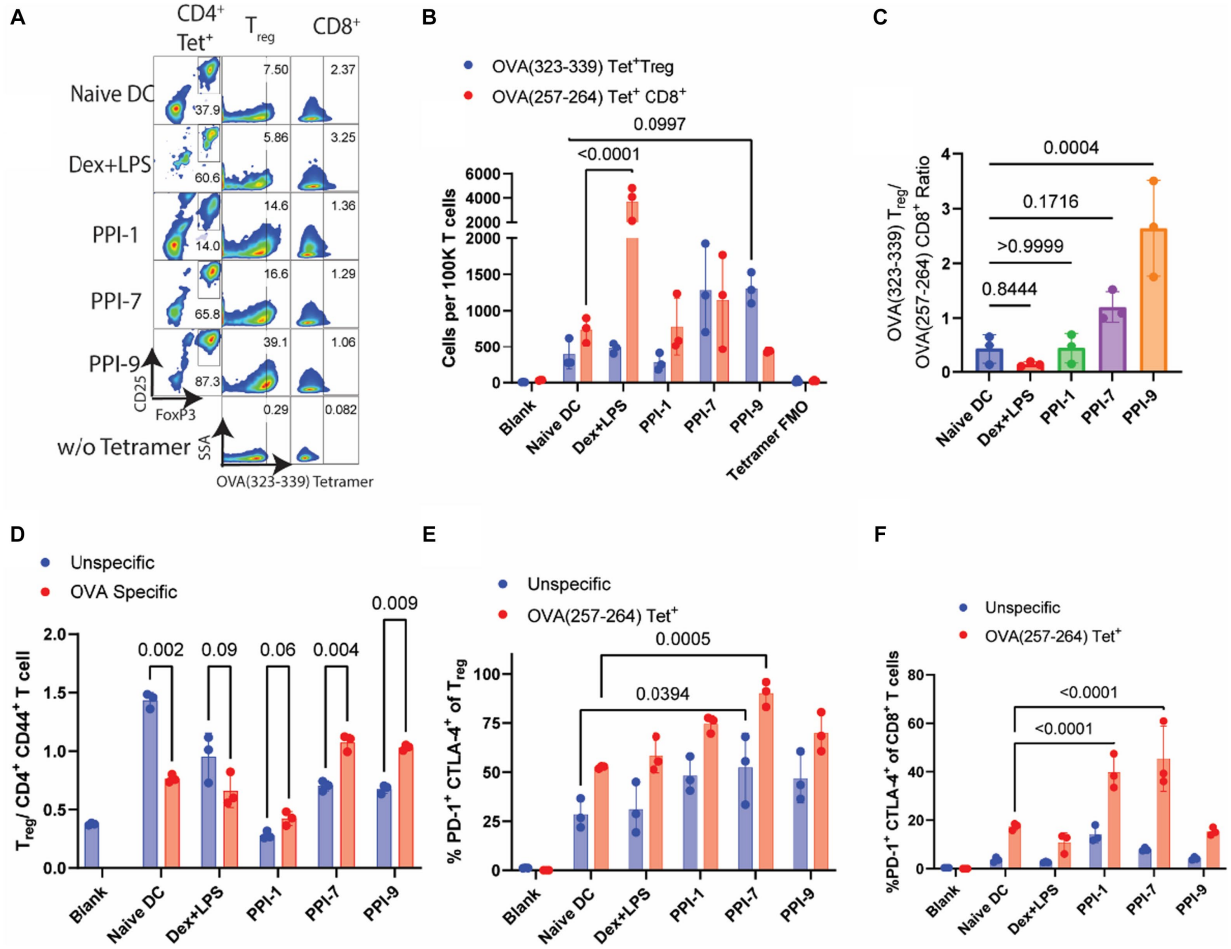
Analysis of OVA-specific T-cell responses from PPI-tolDC-treated T cells. The same groups for both unspecific (αCD3/CD28 simulated) and specific (OVA-stimulated) T cells from Figure 5 were further analyzed for OVA tetramer-positive populations. **(A)** Representative flow plots of OVA T-cell experiment. Left: OVA (323–339)-PE tetramer^+^ CD4^+^ T cells gated on CD25 and FoxP3 for T_reg_ populations; Middle: T_reg_ cell populations gated OVA (323–339)—PE tetramer^+^ populations; Right: CD8^+^ T-cell populations gated on OVA (257–264)—APC tetramer^+^. **(B)** Comparison of OVA (323–339)—PE tetramer^+^ T_reg_ cells and OVA (257–264)—APC tetramer^+^ CD8^+^ T cells per 100K T cells for OVA-stimulated samples. **(C)** Ratios of OVA (323–339)—PE tetramer^+^ T_reg_ cells by OVA (257–264)—APC tetramer^+^ CD8^+^ T cells per 100K T cells. **(D)** Comparison of OVA (323–339)—PE tetramer^+^ Treg cells and OVA (323–339)—PE tetramer^+^ CD4^+^ CD44^+^ T effector cells per 100K T cells. **(E)** Percentage of PD-1^+^ CTLA-4^+^ cells in either all T_regs_ (blue) or OVA (323–339)—PE tetramer^+^ T_regs_ (red). (F) Percentage of PD-1^+^ CTLA-4^+^ cells in either all CD8^+^ T cells (blue) or OVA (257–264)—APC tetramer^+^ CD8^+^ T cells (red). Gating strategies, including tetramers, are found in Supplementary Figure S8. All experiments in this figure were carried out in biological triplicate, and error bars in part E represent the ±SD of triplicate measurements. Significance was calculated using one-way ANOVA with Tukey post-hoc test for parts **(A,B,F)**. All other significance was determined by two-tailed student’s *t*-test.

We similarly evaluated PPI-tolDCs using T cells from OVA-vaccinated T cells in a similar experiment as in Figure 4A and analyzed them using a similar immunophenotyping panel with OVA-specific tetramers (see Supplementary Figure S8 for gating strategies). Here we observed a sharp contrast between antigen-specific and all T_reg_ responses. No PPI-tolDCs group significantly increased the overall Treg populations, although PPI-9 had a moderate, albeit insignificant, increase, corroborating our results in Figures 4F, 5C. There was also a similar decrease in CD4^+^ T-cell populations in the Dex-LPS groups, which was not observed in the PPI treatment groups, further reinforcing that Dex-LPS BMDCs were highly suppressive but not antigen-specific (Supplementary Figure S10). We identified OVA-specific T_reg_ populations based on the major MHC-II epitope, OVA (323–339), and OVA-specific CD8^+^ T cells based on the major MHC-I epitope, OVA (257–264) (Figure 6A; see Supplementary Figure S8 for gating strategy on tetramers). We observed that PPI-7 and PPI-9 increased OVA (323–339) tetramer^+^ T_reg_ populations and decreased OVA (257–264) tetramer^+^ CD8^+^ T cells when compared to Dex-LPS (Figure 6B). We calculated the ratio of OVA (323–339) tetramer^+^ T_reg_ populations to OVA (257–264) tetramer^+^ CD8^+^ T cells and showed that PPI-7 and PPI-9 increased the ratio of antigen-specific T_reg_ to CD8^+^ effector cells, although only PPI-9 had a significant increase (Figure 6C). We further analyzed these data, calculating the ratio of T_reg_ to CD4^+^ CD44^+^ T effector cells in both the non-specific and OVA (323–339) tetramer^+^ populations, and observed that only PPI-7 and PPI-9 significantly increased the OVA-specific ratio compared to the non-specific T-cell ratio (Figure 6D). We further analyzed the T_reg_ and CD8^+^ T-cell populations in both the non-specific and OVA-specific compartments for the expression of PD-1 and CTLA-4, both immunosuppressive markers critical for robust immunosuppressive function (23). We observe that PPI-1 and PPI-7 increase PD-1^+^ CTLA-4^+^ populations only in the OVA-specific compartment for both T_reg_ and CD8^+^ (Figures 6E,F). Overall, these results indicate that both PPI-7 and PPI-9 selectively increase antigen-specific T_reg_ populations and decrease antigen-specific CD8^+^ T cells without increasing overall T_reg_, although PPI-7 appears to generate T_reg_ with higher PD-1 and CTLA-4 expression.

## Discussion

This study used high-throughput screening data from a large library of over 40,000 unique combinations of immune agonists and immunomodulators to identify two novel combinations that generate robust tolDCs, which, in turn, facilitate the generation of antigen-specific immunosuppressive responses. Through several screening studies, we systematically identified optimized combinations that modulate BMDC to express high levels of tolerogenic markers such as IL-10 and PD-L1 and low levels of inflammatory markers such as TNF-α and CD80. We further evaluated the PPI-tolDCs for their ability to prevent T-cell proliferation and generate T_reg_ cells. Finally, we observed that two of our combinations, PPI-7 and PPI-9, generate DCs that suppress immunity in an antigen-specific fashion *ex vivo*.

One aspect of this screening study of note is that the PPI-1 formulation (cucurbitacin I + LPS) generated a very high level of IL-10 but still did not generate a high antigen-specific T_reg_/T_eff_ ratio, like PPI-7 and PPI-9. IL-10 is a widely studied cytokine for tolerance and is often used as a metric for highly tolerating autoimmune therapies, as it was in this study (24, 25). A more robust measure of antigen-specific immune suppression and clinical potential, however, is the downstream antigen-specific T_reg_/T_eff_ ratio (26). This is part of a larger question about what markers on tolDCs indicate they will generate robust antigen-specific T_reg_ responses. While IL-10 generation and the antigen-specific T_reg_/T_eff_ ratio are correlated, here we observe that PPI-7 and PPI-9 (formulations with the highest antigen-specific T_reg_/T_eff_ ratio), while mildly increasing IL-10, at least did not suppress PD-L1. PD-L1 is commonly studied in cancer immunotherapy, as PD-L1 inhibition improves some cancer therapies and has been shown to be important for tolerating cell phenotypes (27, 28). Furthermore, PPI-7 and PPI-9 only moderately influenced CD40 and CD80 markers on DCs but still highly suppressed TNF-α secretion. This suggests that some CD40 and CD80 may be beneficial to generating robust tolDCs, and IL-10 expression alone is not sufficient for predicting the efficacy of a tolDC therapy. With such a small sample, we cannot draw any definitive conclusions on which markers indicate potent tolDCs, but our data are encouraging for further study.

Another encouraging result of this study is the identification of three immunomodulatory compounds that warrant future study in generating tolDCs. Our top three PPIs contained cucurbitacin I, AD-80, and CEP-33779. Cucurbitacins are tetracyclic triterpene compounds found in melons, cucumbers, and pumpkins; these are known to have anti-inflammatory effects (29). These molecules, including cucurbitacin I, have been shown to have anti-cancer properties as a JAK inhibitor, and cucurbitacin B was used in a study prophylactically to prevent a mouse model of multiple sclerosis (29, 30). Similarly, CEP-33779, is also a JAK inhibitor, known to prevent cellular maturity and has been used in autoimmune models as a non-specific immunomodulator (31, 32). AD-80 (from PPI-7), meanwhile, is a multi-kinase inhibitor known to modulate PI3K and RAF and, to date, has only been investigated for anti-cancer properties (33). It is well established that interfering with kinase activity can prevent inflammation, as immune activation and maturity are governed by a complex network of kinase activity. To date, many anti-inflammatory kinase inhibitor drugs have been brought to clinical trials (34). It is unsurprising that kinase inhibitors generate robust tolDCs as important kinases such as PI3K have been implicated in tolDC development previously (35). This study was the first to systematically identify three unique kinase modulators that generate tolDCs with potent Treg-influencing behavior.

This study did have notable limitations. First, we used exclusively murine BMDCs and did not employ human cells. Second, all experiments were performed *in vitro*. Both factors, while increasing reproducibility and reducing cost, also reduced the direct clinical impact of the study. Finally, this study did not uncover the mechanism by which these PPI combinations work synergistically or which cellular mechanisms are modified.

We plan to address all these limitations in future research. Specifically, we plan to first test the identified PPI candidates on human monocyte-derived DCs, which are the typical mechanism for generating tolDCs in the clinic. We will ensure that our PPI compounds generate tolDC phenotypes and further characterize the PPI-tolDCs. We will also perform animal models of autoimmunity and transplant to ensure that our PPI-tolDCs can generate not only antigen-specific T_regs_, but also functional T_regs_ that can suppress allo/autoimmunity. Our primary goal of this study, meanwhile, was to identify a handful of viable new PPI candidates from a large dataset, which was accomplished. Overall, we present an exciting new strategy for generating tolDCs *ex vivo* and highlight the potential for PPI-tolDC to be further refined into a clinical therapeutic for autoimmune diseases.

## Data availability statement

The original contributions presented in the study are included in the article/Supplementary material, further inquiries can be directed to the corresponding author.

## Ethics statement

Ethical approval was not required for the studies on humans in accordance with the local legislation and institutional requirements because only commercially available established cell lines were used. The animal study was approved by Drexel University Institutional Animal Care and Use Committee. The study was conducted in accordance with the local legislation and institutional requirements.

## Author contributions

SJ: Data curation, Formal analysis, Investigation, Methodology, Validation, Visualization, Writing – original draft, Writing – review & editing. JK: Data curation, Formal analysis, Investigation, Methodology, Software, Visualization, Writing – original draft, Writing – review & editing. AE-K: Conceptualization, Funding acquisition, Resources, Supervision, Writing – review & editing. PD: Conceptualization, Data curation, Formal analysis, Funding acquisition, Investigation, Methodology, Project administration, Resources, Software, Supervision, Validation, Visualization, Writing – original draft, Writing – review & editing.
