# Supplementary material for: High-throughput screening identification of novel immunomodulatory combinations for the generation of tolerogenic dendritic cells
Source: Front Med (Lausanne). 2024 Jan 5;10:1298424. doi: 10.3389/fmed.2023.1298424 (PMC10796829; doi:10.3389/fmed.2023.1298424)
Supplement: Supplementary file 1 [file Data_Sheet_1.PDF]

Supplementary Information for: ***High Throughput Screening Identification of Novel Immunomodulatory Combinations for Generation of Tolerogenic Dendritic Cells***

Jia Sihan[1†], Jeremiah Kim[2†], Aaron Esser-Kahn[2], and Peter Deak[1*]

[1]Chemical and Biological Engineering Department, Drexel University, Philadelphia, PA 19104

[2]Pritzker School of Molecular Engineering, University of Chicago, Chicago, IL 60637

[†] Jia Sihan and Jeremiah Kim contributed equally

*** Correspondence:**

Center for Automation Technologies
3101 Ludlow St, Room 480
Philadelphia, PA 19104
pd562@drexel.edu
215-895-6694

Contents:
Table S1-S3
Figure S1-S10

Table S1: Source libraries for compounds used in primary screen

Library	Source	Number
**Inhibitors**	Selleckchem	2790
**NF-κB Inhibitors**	Selleckchem	41
**NF-κB Inhibitors**	MedChem Express	206
**Tolerance**	Various	104
	**Total**	3141

Table S2. Agonists used in primary screen. Agonist targets, pathways activated, and working concentrations.

Agonist	*Target*	*NF-κB*	*IRF*	*Conc (ug/mL)*
Pam2CSK4	**TLR2/1**	**X**		**0.1**
Pam2CSK4	**TLR2/6**	**X**		**0.1**
Poly I:C	**TLR3**		**X**	**1**
MPLA	**TLR4**	**X**	**X**	**0.25**
LPS	**TLR4**	**X**	**X**	**0.1**
Flagellin	**TLR5**	**X**	**X**	**1**
Imiquimod	**TLR7**	**X**	**X**	**2.5**
TL8-506	**TLR8**		**X**	**0.1**
ODN 1826	**TLR9**	**X**	**X**	**0.5**
3'3'-cGAMP	**STING**		**X**	**10**
Tri-DAP	**NOD1**	**X**		**25**
MDP	**NOD2**	**X**		**10**
mIFN-β	**IFNAR1/2**	**X**	**X**	**0.00833**

Table S3. Top 8 PPI that increased IL-10 and PD-L1 normalized to agonist only

Ratio (Mod/Agonist)	PPI	IL-10+PD-L1 (Normalized)
0.1:0.1	1	21.7
0.1:1	1	21.1
0.1:0.1	8	5.2
0.1:1	8	4.6
0.1:1	7	3.7
0.1:1	3	2.7
0.1:1	5	2.5
0.1:1	9	2.5
1:0.1	8	2.3
0.1:0.1	7	2.3
1:0.1	5	2.2
0.1:0.1	9	2.1
0.1:0.1	6	2.0
0.1:1	6	1.9
0.1:0.1	3	1.9
0.1:0.1	5	1.7
1:0.1	Dex+LPS	1.7
1:1	8	1.7
0.1:0.1	Dex+LPS	1.7
0.1:1	10	1.7
1:1	5	1.7
0.1:1	2	1.5
0.1:1	Dex+LPS	1.4
1:1	Dex+LPS	1.4
0.1:1	4	1.2
0.1:0.1	10	1.0
1:1	6	1.0
1:0.1	6	1.0
0.1:0.1	2	1.0
0.1:0.1	4	0.9
1:1	9	0.9
1:1	10	0.8
1:0.1	9	0.8
1:0.1	10	0.6
1:1	3	0.5
1:1	7	0.4
1:1	2	0.3
1:1	1	0.3
1:1	4	0.3
1:0.1	1	0.3
1:0.1	2	0.3
1:0.1	3	0.2
1:0.1	7	0.1
1:0.1	4	0.0

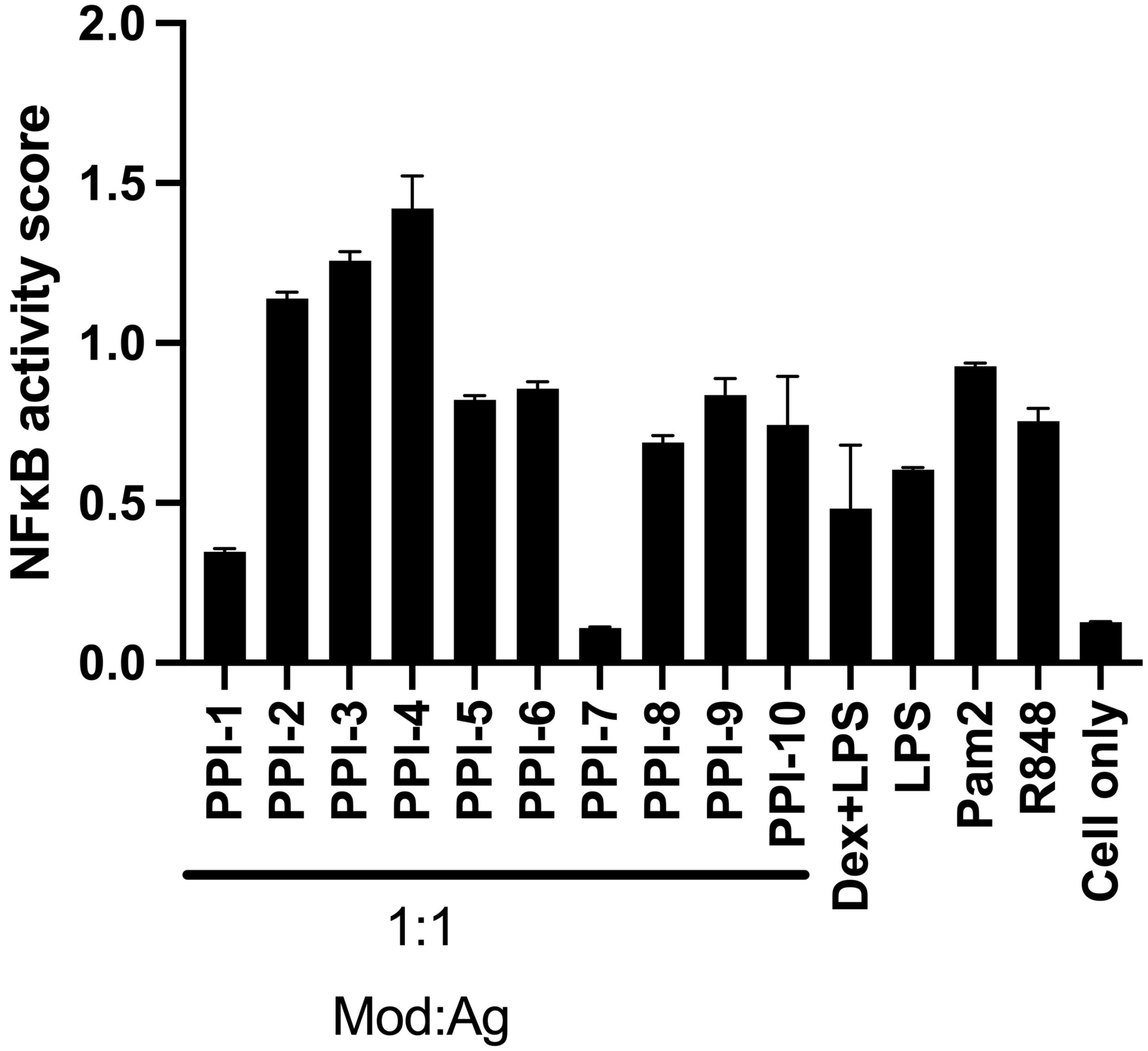

Figure S1. PPI effect on NF-κB transcriptional activity. The 1:1 modulator and agonist ratio of the top 10 PPI combinations identified in table 1 were incubated with 100k Raw Blue cells for 24 h and then tested via Quanti-Blue Assay to track PPI effect on NF-κB transcriptional activity. Cells were tested in biological triplicates; error bars indicate +/- SD.

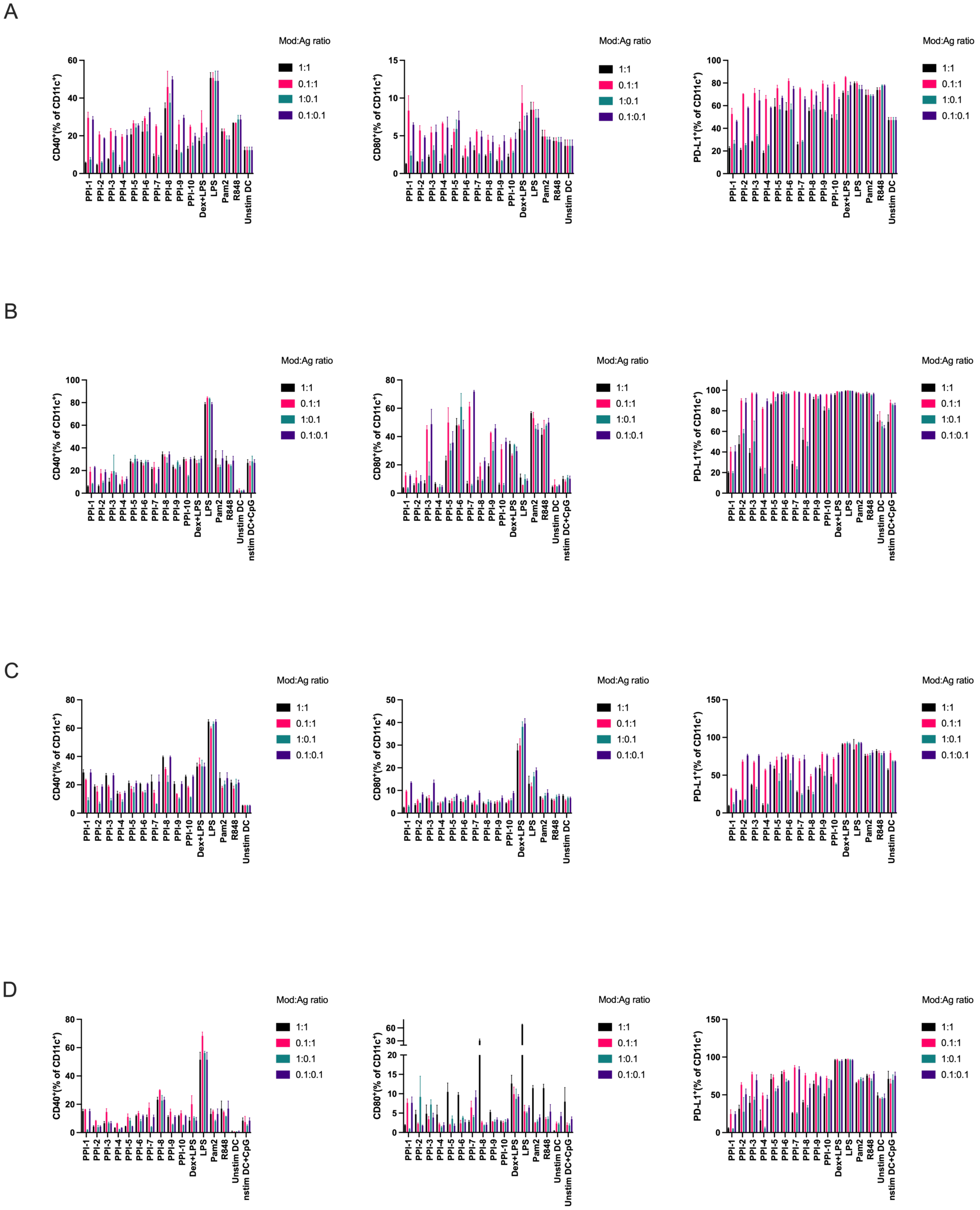

Figure S2. CD40, CD80, PD-L1 expression of top 10 PPIs. The modulator and inhibitor ratios of the top 10 PPI combinations identified in figure 1 were altered to generate four different PPIs (1:1, 0.1:1, 1:0.1 and 0.1:0.1 modulator to agonist ratio). PPIs were incubated with 200K BMDCs both on day 5 or 10 for 24 h and either tested directly or treated with 0.5µM CpG for another 24 h, then tested for CD40, CD80, PD-L1 expression(left to right) via flow cytometry. A) CD40, CD80, PD-L1 expression for BMDCs incubated with PPIs on day 5 with CpG treatment. B) CD40, CD80, PD-L1 expression for BMDCs incubated with PPIs on day 5 without CpG treatment. C) CD40, CD80, PD-L1 expression for BMDCs incubated with PPIs on day 10 with CpG treatment. D) CD40, CD80, PD-L1 expression for BMDCs incubated with PPIs on day 10 without CpG treatment. N=3 and error bars indicate SD.

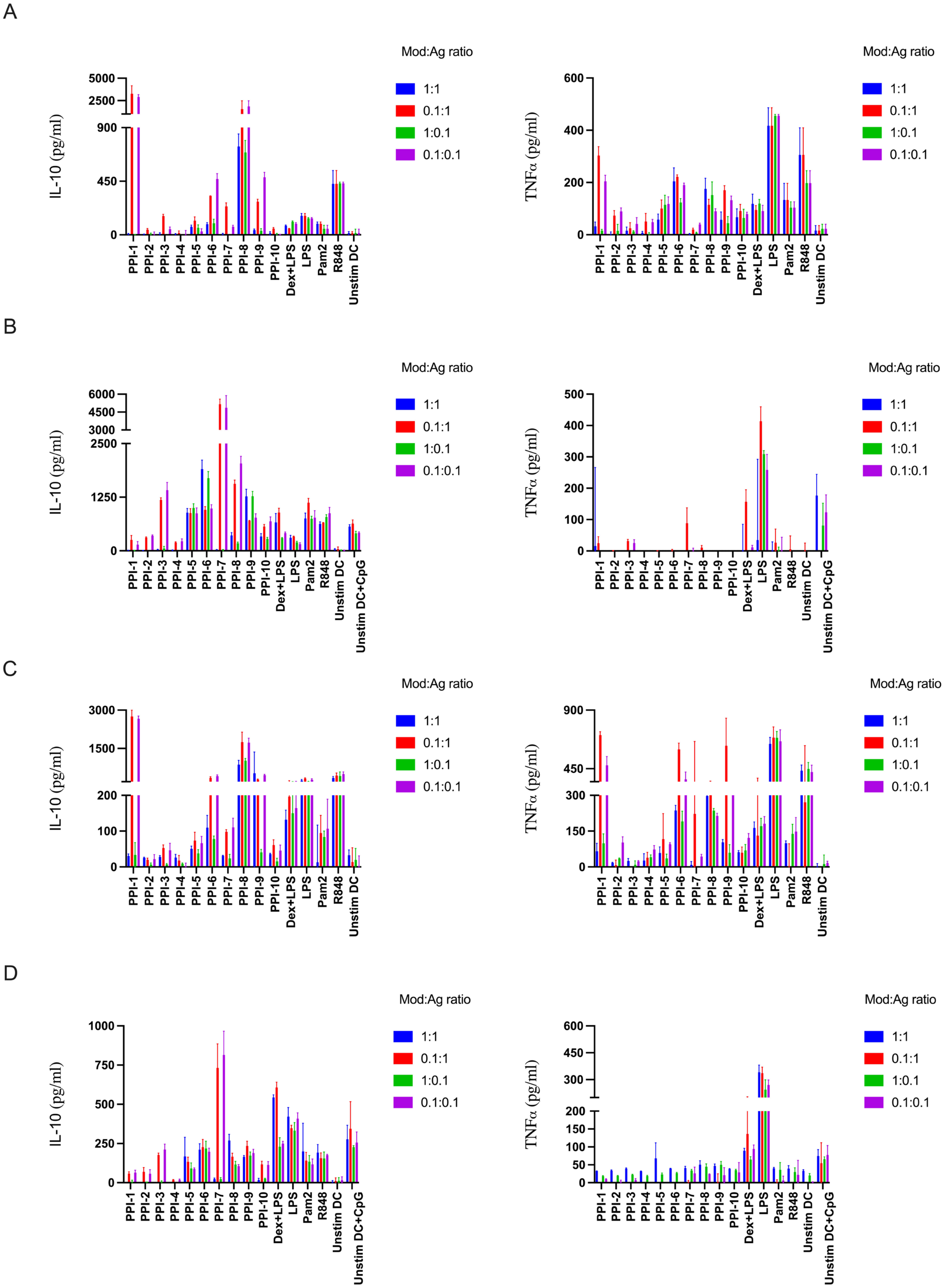

Figure S3. Cytokine Secretion of top 10 PPIs tested on BMDCs. The modulator and inhibitor ratios of the top 10 PPI combinations identified in figure 1 were altered to generate four different PPIs (1:1, 0.1:1, 1:0.1 and 0.1:0.1 modulator to agonist ratio). PPIs were incubated with 200K BMDCs both on day 5 or 10 for 24 h and either tested directly or treated with 0.5µM CpG for another 24 h, then tested for IL-10 and TNF-α(left to right) secretion via ELISA. A) IL-10 and TNF-α secretion for BMDCs incubated with PPIs on day 5 without CpG treatment. B) IL-10 and TNF-α secretion for BMDCs incubated with PPIs on day 5 with CpG treatment. C) IL-10 and TNF-α secretion for BMDCs incubated with PPIs on day 10 without CpG treatment. D) IL-10 and TNF-α secretion for BMDCs incubated with PPIs on day 10 with CpG treatment. N=3 and error bars indicate SD.

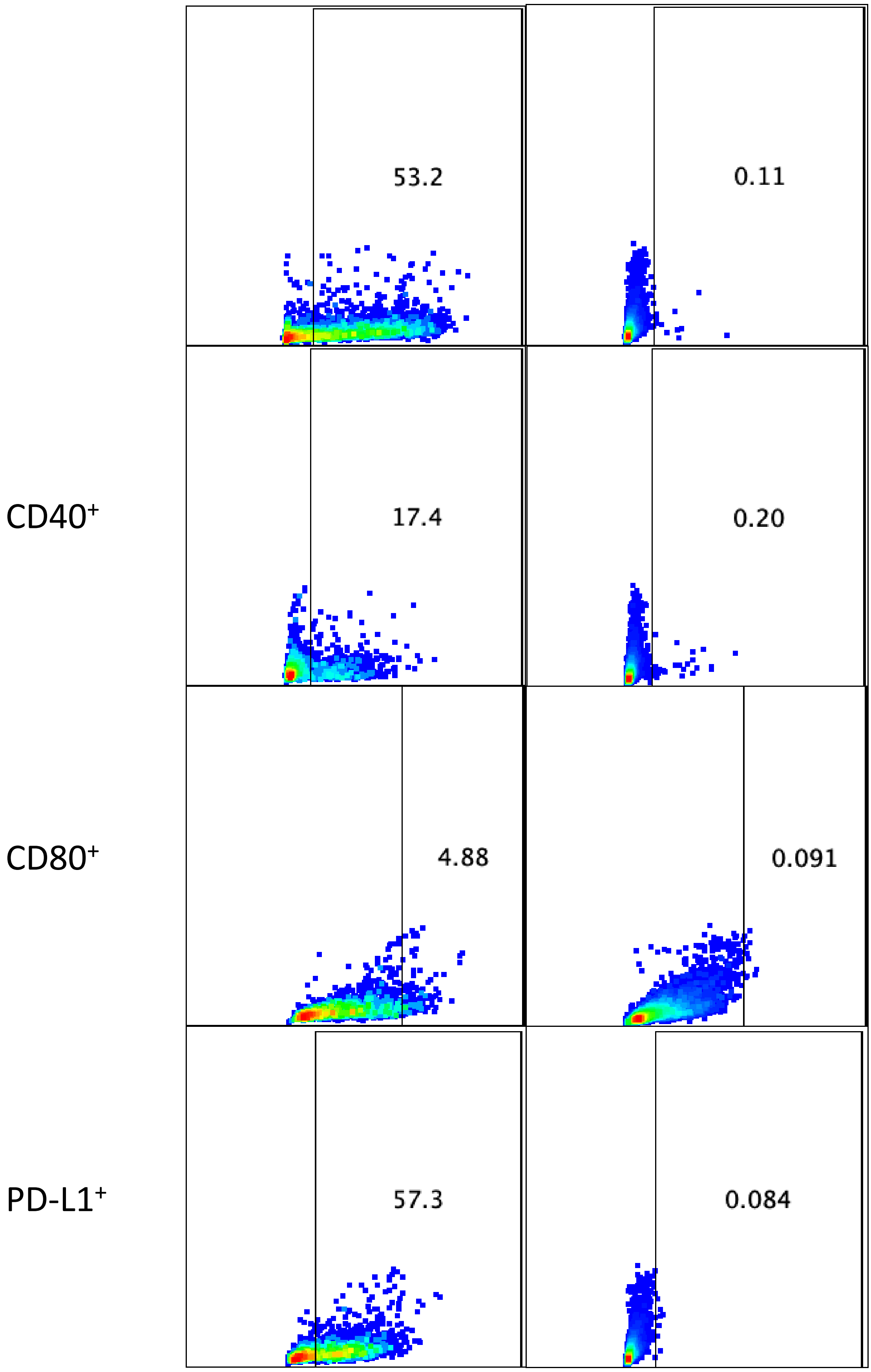

Figure S4. Gating strategy for BMDCs in Figure 2. Gating of positive vs negative populations for four cell markers: CD11c, CD40, CD80 and PD-L1(top to bottom). Left- Stained samples, Right- unstained samples.

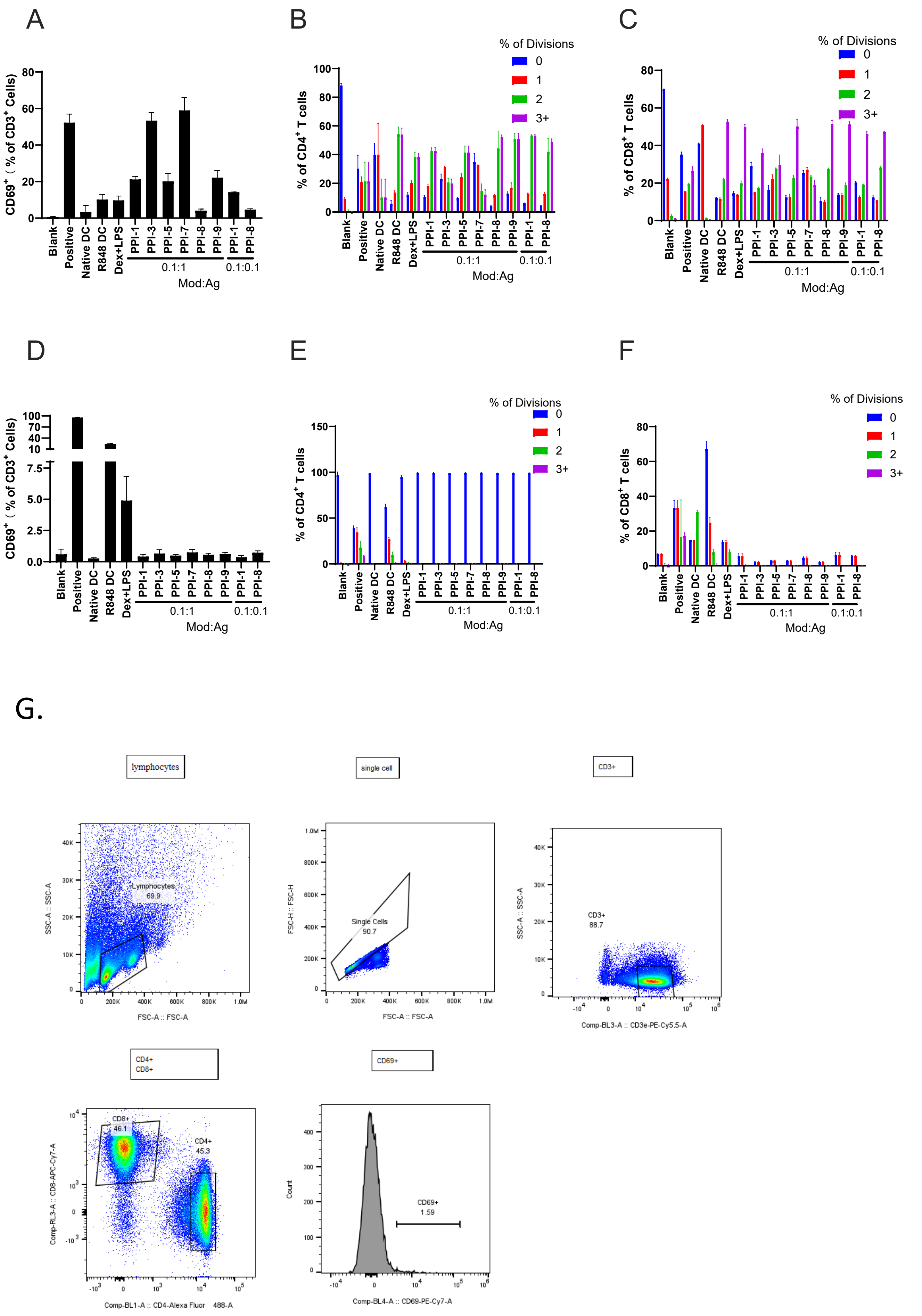

Figure S5. Additional Data for T cell stimulation assay using Top 8 PPI combinations. (A-C) T cells were stimulated as in Figure 3. 800K T cells were incubated with 200K DCs and analyzed after 72 h. Controls included: Blank= T cell only; Positive= T cells+ 0.5 µL/mL T cell stimulation cocktail with PMA/ionomycin; Naive DC= Untreated BMDCs. All groups contained T cells and all groups except blank were treated with 100 ng/mL αCD28/CD3 (A) CD69 expression as measured by flow cytometry. B) CFSE staining for T cell proliferation, gated on CD4+ T cells. C) CFSE staining for T cell proliferation, gated on CD8+ T cells. (D-F) T cells were stimulated for OVA specific activation as in Figure 4. 800K T cells from OVA vaccinated mice were incubated with 200K DCs for each group and analyzed after 72 h. OVA vaccination was 1 mg/mL. Controls included: Blank = T cells from OVA vaccinated mice only; Positive = T cells from OVA vaccinated mice + naïve BMDCs + 0.5 µL/mL T cell stimulation cocktail with PMA/ionomycin; Naive DC = T cells from OVA vaccinated mice + naïve BMDCs. All groups except Blank were treated with 10 µg/mL OVA. (D) CD69 expression as measured by flow cytometry. E) CFSE staining for T cell proliferation, gated on CD4+ T cells. F) CFSE staining for T cell proliferation, gated on CD8+ T cells. G) Representative gating strategy. All controls samples were performed in duplicates and experimental groups in biological triplicates. Error bars are ± of SD.

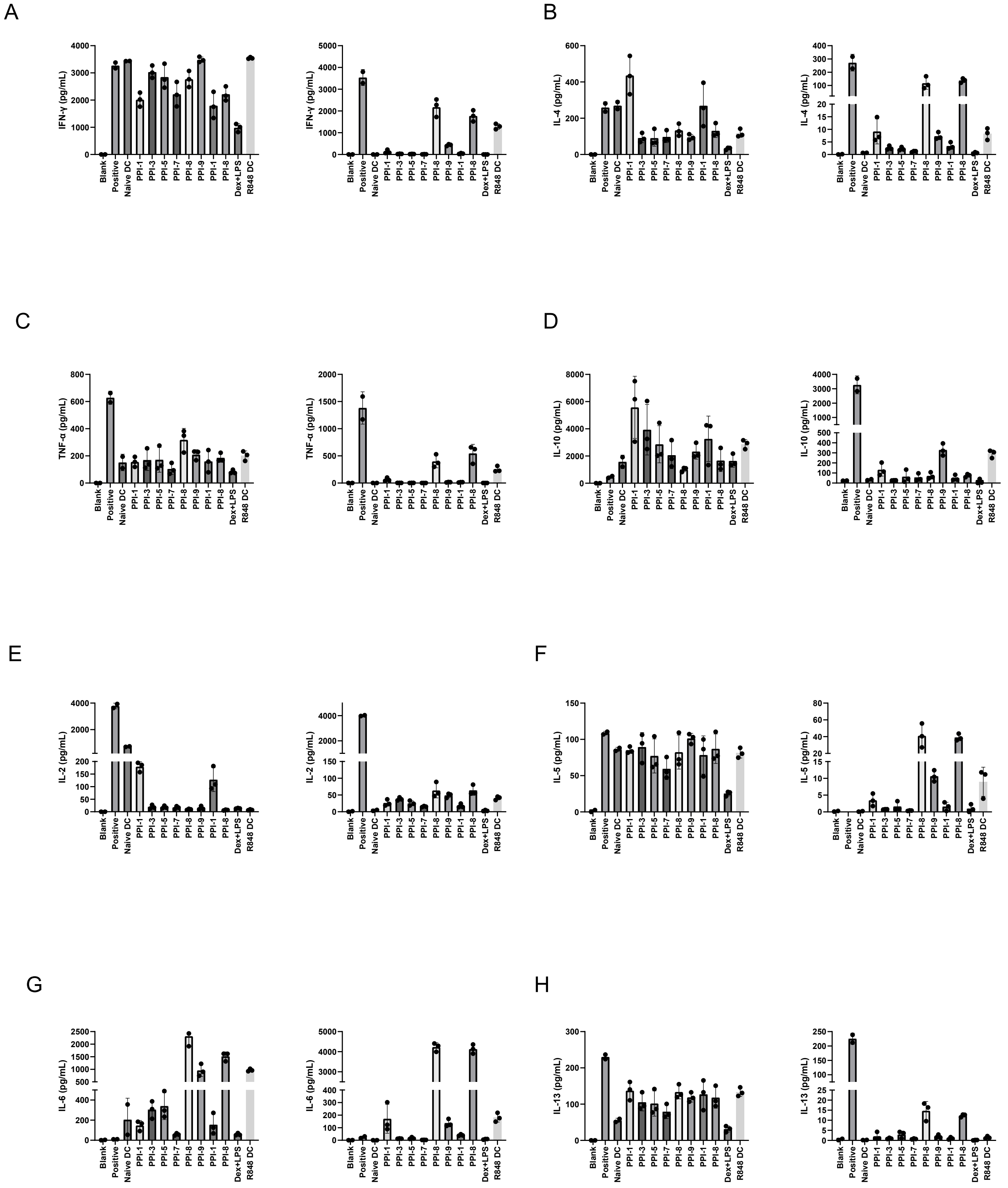

Figure S6. Cytokine secretion of top 8 PPIs tested on T cells. In the non-specific assay (left for each subplot), 800K T cells were incubated with 200K DCs. Controls included: Blank= T cell only; Positive= T cells+ 0.5 µL/mL T cell stimulation cocktail with PMA/ionomycin; Naive DC= Untreated BMDCs. All groups contained T cells and all groups except blank were treated with 100 ng/mL αCD28/CD3. In the OVA specific assay (right for each subplot), 800K T cells from OVA vaccinated mice were incubated with 200K DCs for each group. OVA vaccination was 1 mg/mL. Controls included: Blank = T cells from OVA vaccinated mice only; Positive = T cells from OVA vaccinated mice + naïve BMDCs + 0.5 µL/mL T cell stimulation cocktail with PMA/ionomycin; Naive DC = T cells from OVA vaccinated mice + naïve BMDCs. All groups except Blank were treated with 10 µg/mL OVA. For both assays, tested for IFN-γ(A), IL-4(B), TNF-α(C), IL-10(D), IL-2(E), IL-5(F), IL-6(G) and IL-13(H) secretion after incubation for 72h. All controls samples were performed in duplicates and experimental groups in biological triplicates. Error bars are ± of SD.

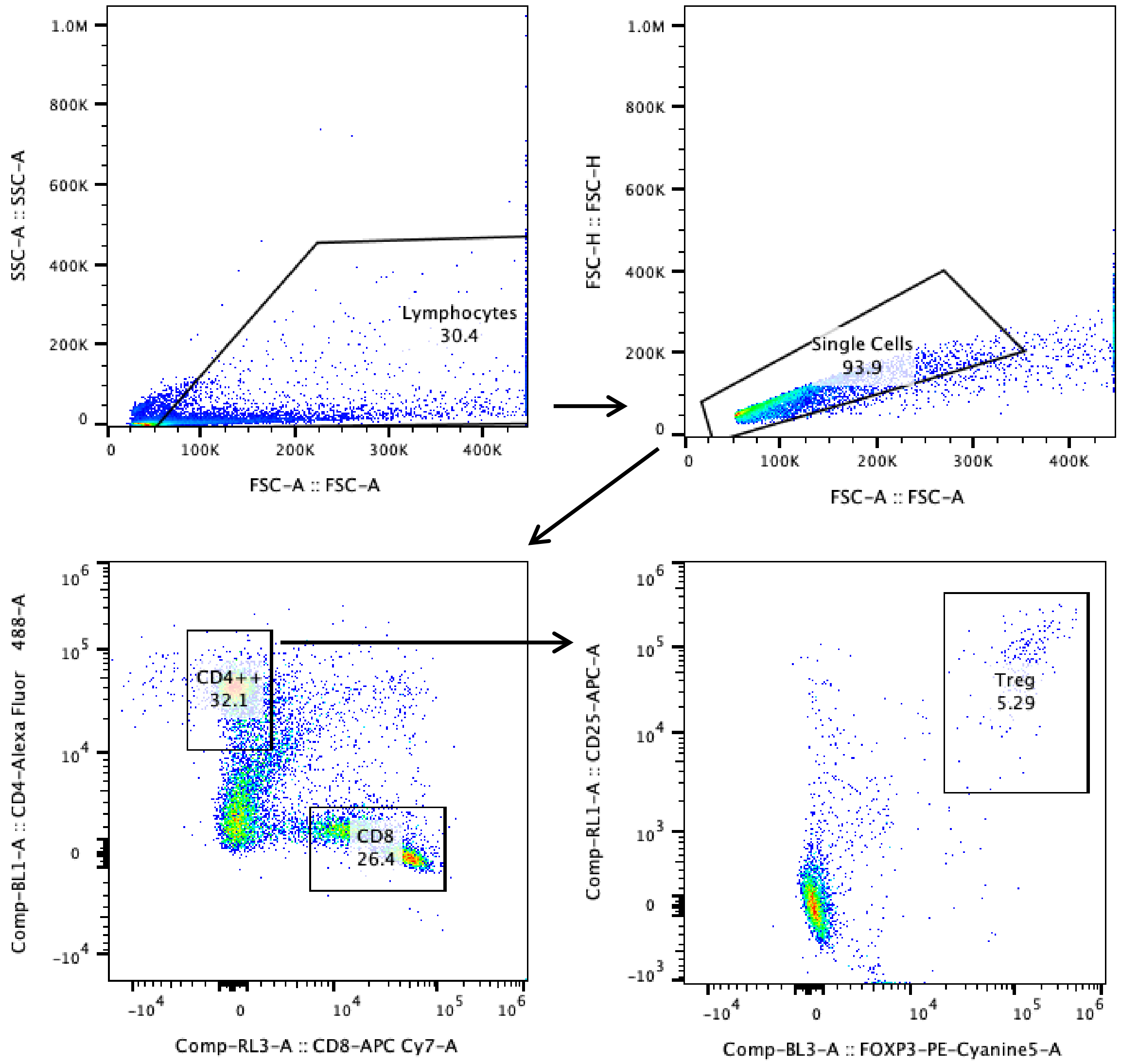

Figure S7. Gating strategy for T_{reg} cells in Figure 3 and 4. General gating strategy of T_{reg} cells- (Lymphocytes, Single Cells, CD4+/CD8-, CD25+/FoxP3+).

A.

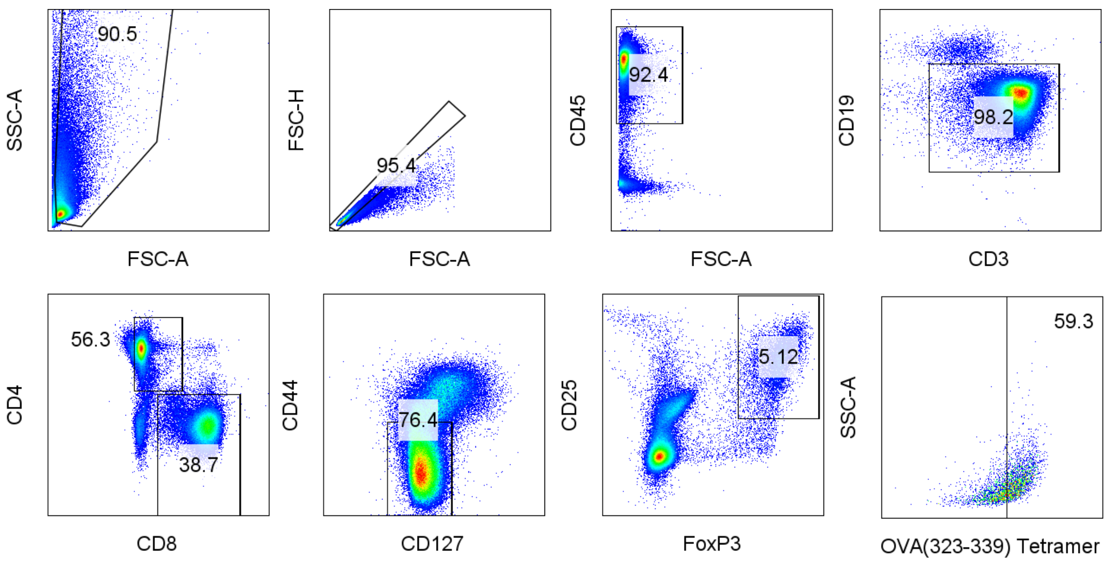

B.

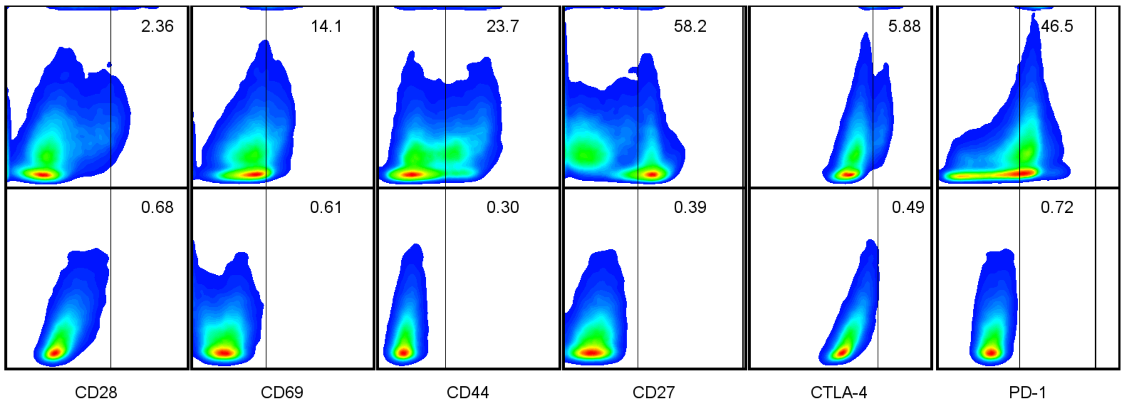

C.

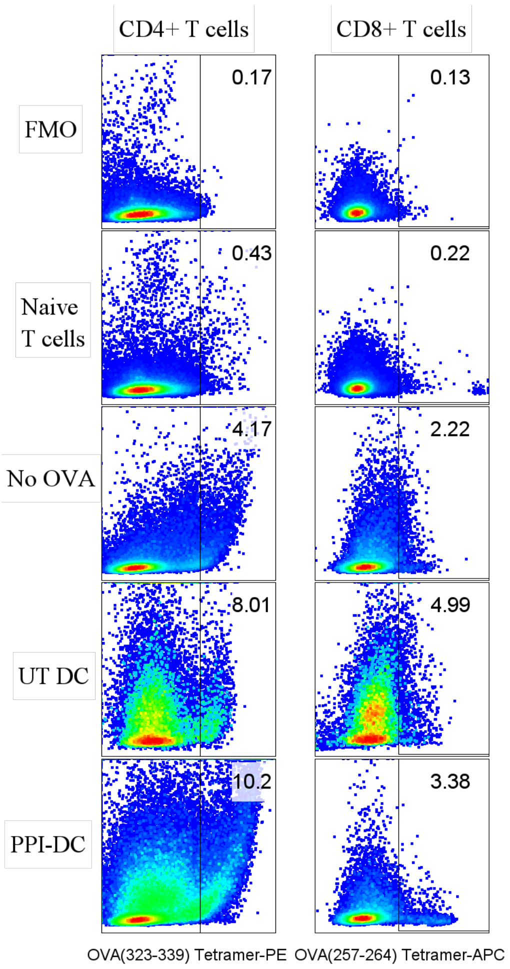

Figure S8. Gating Strategy for Figure 5/6. Gating of large immunophenotyping panel with tetramers. (A) General gating strategy of Treg cells- (Live, Single Cells, CD45+, CD19-/CD3-, CD4+/CD8-, CD44-/CD127-, CD25+/FoxP3+). (B) Gating of positive vs negative populations for various markers. Top- Stained samples, Bottom- unstained samples. (C) Gating strategy for tetramer-stained populations on CD3+ CD45+ OVA sensitized cells. Left- OVA(323-339)-PE on CD4+ T cells and Right- OVA(257-264)-APC on CD8+ T cells with various control populations. FMO= Flow minus one, UT- untreated DCs. PPI-DCs- cells treated with PPI-9

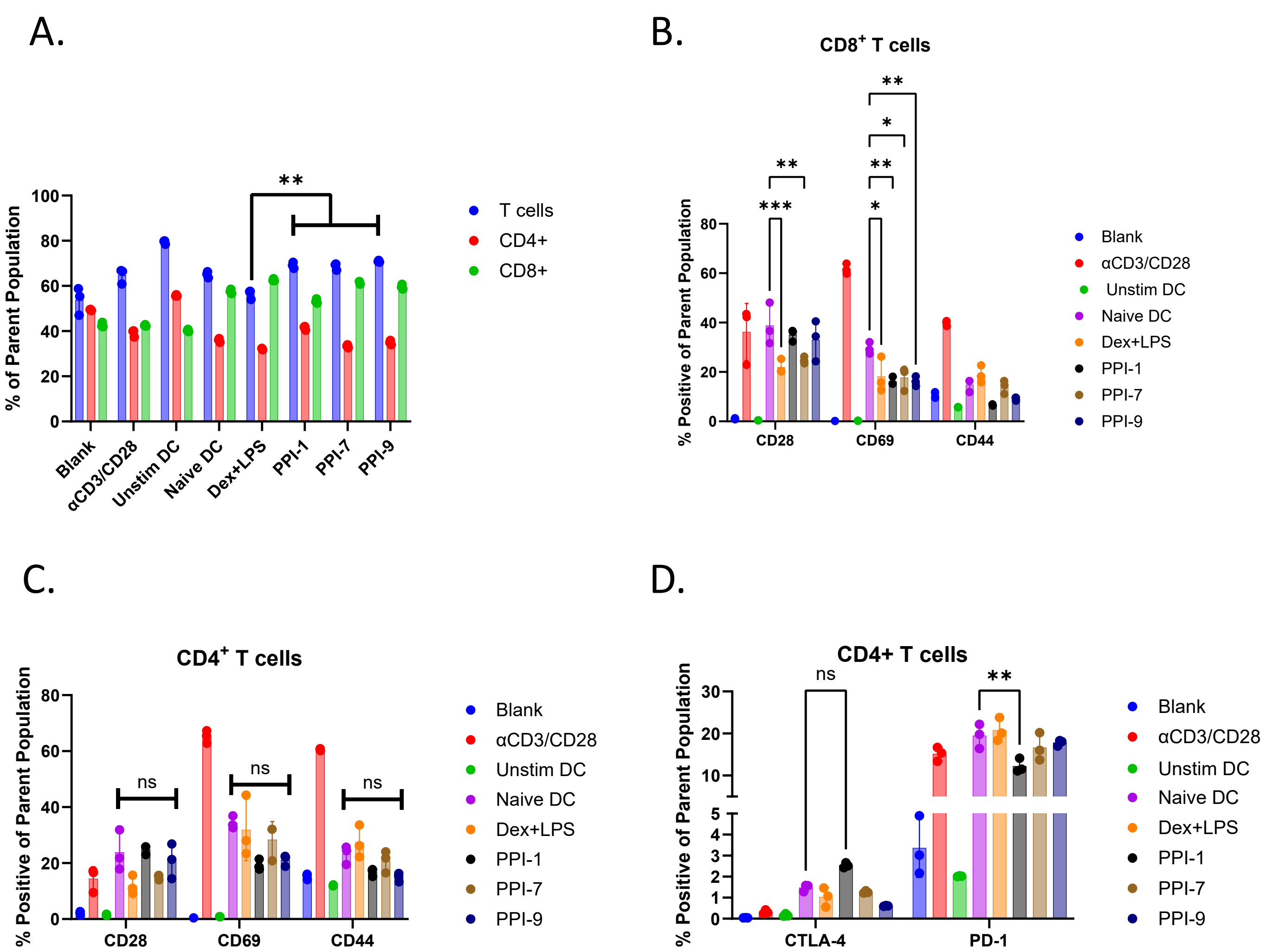

Figure S9. Additional data from non-specific T cell analysis in Figure 5 and 6. All cells were taken from naïve C57Bl/6 mice and T cell stimulated with αCD3/CD38 antibodies. (A) PPI treatment does not impair T cell viability. Cell breakdown of both total CD3+ T cells (of live CD45+) and CD4+/CD8+ T cells. Note Dex+LPS treated BMDCs significantly reduce total T cell populations. (B) % CD28, CD69 and CD44+ cells as percent of total CD8 T cells. (C) % CD28, CD69 and CD44+ cells as percent of total CD4 T cells. (D) % CTLA-4 and PD-1+ cells as percent of total CD4+ T cells. Significance was calculated using one way ANOVA with Tukey post hoc test. *p<0.05, **p<0.01, ***p<1x10-4

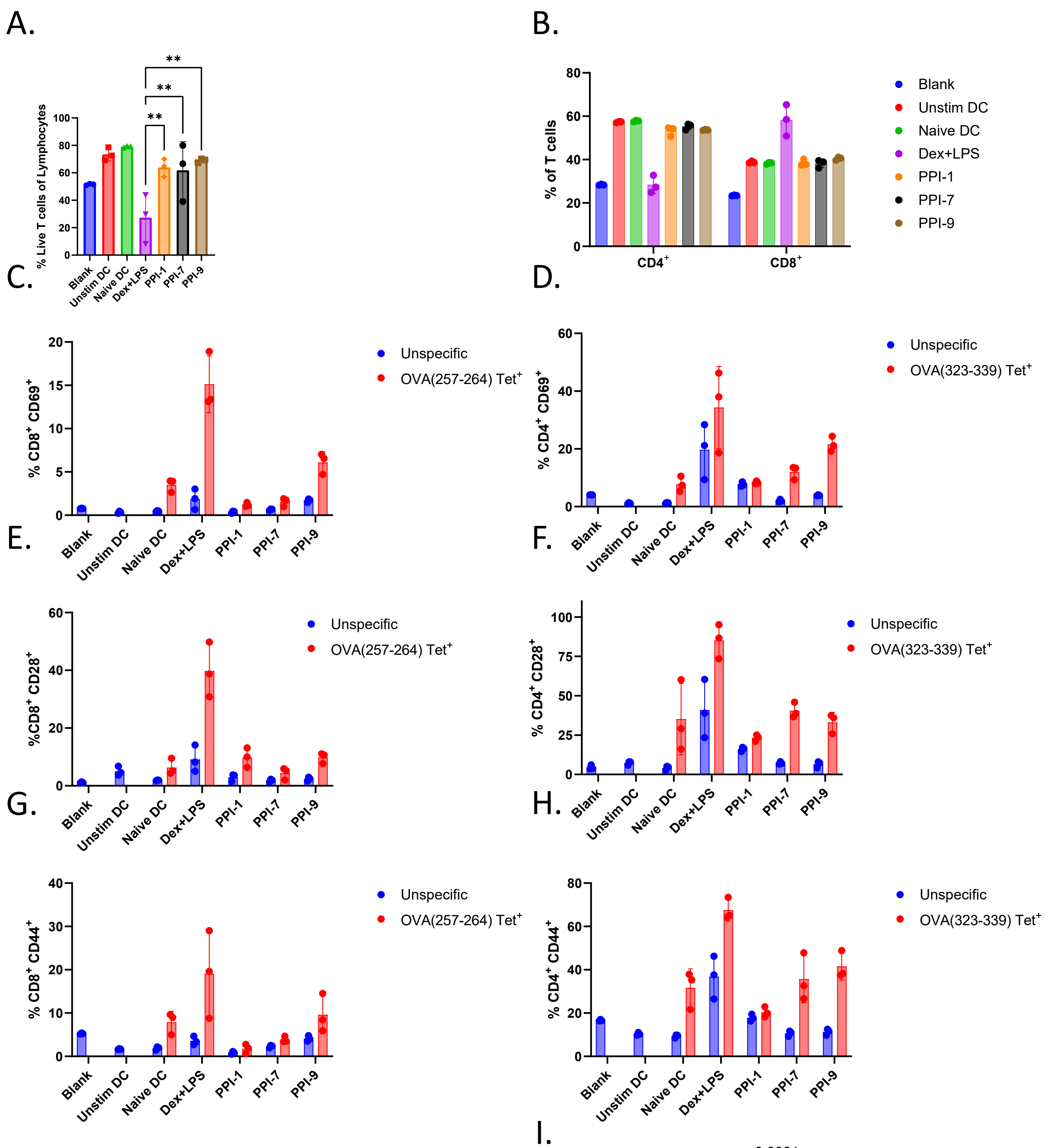

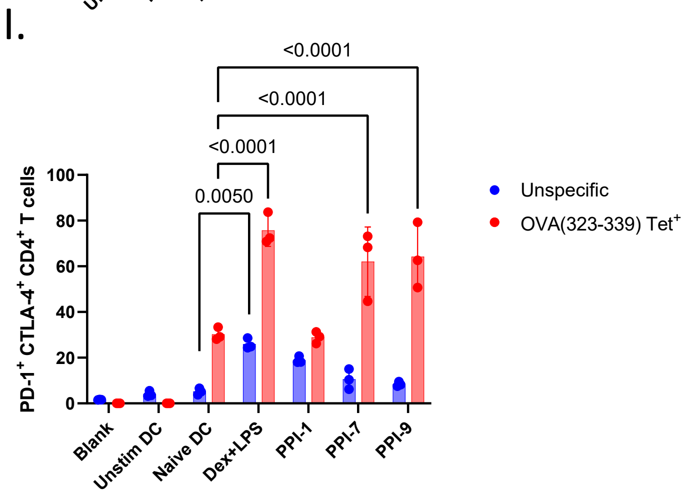

Figure S10. Additional data from cell analysis in Figure 5 and 6. (A) PPI treatment does not impair T cell viability of OVA sensitized T cells. Cell breakdown live CD3+ CD45+ as percent of lymphocytes. Note Dex+LPS treated BMDCs significantly reduce total T cell populations. (B) PPI-tolDCs do not impact CD4/CD8 t cell ratios of cells from part A, as percent of all CD3+ cells. (C-H) Direct comparison of effect of PPI-tolDCs on OVA specific or non-specific T cell simulation. Blue bars indicate CD3/CD28 simulated T cells from naïve mice, red bars indicate OVA simulated T cells from OVA sensitized mice. All populations were live, CD45+ CD3+ T cells. All red bars are either OVA tetramer specific, either CD8+ OVA 257-264) or CD4+ OVA(323-339). (C) % CD69+ of CD8+ T cells, (D) % CD69+ of CD4+ (E) % CD28+ of CD8+ T cells, (F) % CD28+ of CD4+ (G) % CD44+ of CD8+ T cells, (H) % CD44+ of CD4+ T cells. (I) % PD-1, CTLA-4+ of CD4+ Significance was calculated using one way ANOVA with Tukey post hoc test. *p<0.05, **p<0.01, ***p<1x10-4
